# The Role of Pontin and Reptin in Cellular Physiology and Cancer Etiology

**DOI:** 10.3389/fmolb.2017.00058

**Published:** 2017-08-24

**Authors:** Yu-Qian Mao, Walid A. Houry

**Affiliations:** ^1^Department of Biochemistry, University of Toronto Toronto, ON, Canada; ^2^Department of Chemistry, University of Toronto Toronto, ON, Canada

**Keywords:** Pontin, Reptin, AAA+, cancer, cellular pathways

## Abstract

Pontin (RUVBL1, TIP49, TIP49a, Rvb1) and Reptin (RUVBL2, TIP48, TIP49b, Rvb2) are highly conserved ATPases of the AAA+ (ATPases Associated with various cellular Activities) superfamily and are involved in various cellular processes that are important for oncogenesis. First identified as being upregulated in hepatocellular carcinoma and colorectal cancer, their overexpression has since been shown in multiple cancer types such as breast, lung, gastric, esophageal, pancreatic, kidney, bladder as well as lymphatic, and leukemic cancers. However, their exact functions are still quite unknown as they interact with many molecular complexes with vastly different downstream effectors. Within the nucleus, Pontin and Reptin participate in the TIP60 and INO80 complexes important for chromatin remodeling. Although not transcription factors themselves, Pontin and Reptin modulate the transcriptional activities of *bona fide* proto-oncogenes such as MYC and β-catenin. They associate with proteins involved in DNA damage repair such as PIKK complexes as well as with the core complex of Fanconi anemia pathway. They have also been shown to be important for cell cycle progression, being involved in assembly of telomerase, mitotic spindle, RNA polymerase II, and snoRNPs. When the two ATPases localize to the cytoplasm, they were reported to promote cancer cell invasion and metastasis. Due to their various roles in carcinogenesis, it is not surprising that Pontin and Reptin are proving to be important biomarkers for diagnosis and prognosis of various cancers. They are also current targets for the development of new therapeutic anticancer drugs.

## Introduction

Pontin (RUVBL1, TIP49, TIP49a, Rvb1) and Reptin (RUVBL2, TIP48, TIP49b, Rvb2) belong to the AAA+ (ATPases Associated with various cellular Activities) superfamily whose proteins are characterized by having the conserved Walker A and Walker B motifs, which are involved in ATP binding and hydrolysis (Grigoletto et al., 2011; Matias et al., 2015). Pontin and Reptin were discovered in the late 1990s in a variety of species by multiple groups, resulting in their different naming conventions. The proteins are also putative DNA helicases, sharing homology with the bacterial RuvB helicase (Otsuji et al., 1974; Makino et al., 1998; Kurokawa et al., 1999). However, their function as helicases is not yet established and remains controversial. There are also debates in regards to their oligomeric state as they have been observed to form homo-hexamers, hetero-hexamers, and even hetero-dodecamers (Matias et al., 2006; Cheung et al., 2010a; Niewiarowski et al., 2010; Gorynia et al., 2011). It is also likely that Pontin and Reptin assume different oligomeric states under different functional contexts based on their cellular activities (Grigoletto et al., 2011; Nano and Houry, 2013). For example, Queval et al. (2014) proposed that the oligomerization of Pontin and Reptin can be controlled by interaction of the proteins with the nucleosome.

The Pontin/Reptin cellular activities include: transcriptional regulation, chromatin remodeling, DNA damage signaling and repair, assembly of macromolecular complexes, regulating cell cycle/mitotic progression, and cellular motility, all of which contribute to their central roles in promoting cell proliferation and survival (Gallant, 2007; Jha and Dutta, 2009; Boulon et al., 2012; Nano and Houry, 2013; Rosenbaum et al., 2013; Kakihara and Saeki, 2014). This also makes them ideal candidates for promoting tumorigenesis and cancer development, especially when activating mutations occur upstream or downstream in their functional pathways (Grigoletto et al., 2011; Matias et al., 2015; Zhao et al., 2015). Not surprisingly, Pontin and Reptin were shown to be essential for tumor cell growth of many cancers and were found to be overexpressed in a large number of cancer types. Thus, here we will summarize the cancer cell types that Pontin and Reptin are involved in and explore the molecular pathways in which Pontin and Reptin contribute to oncogenesis.

## Roles of pontin/reptin in cancer

The role of Pontin and Reptin in the development of hepatocellular carcinoma (HCC) is well-established (Haurie et al., 2009; Berasain, 2010; Menard et al., 2010; Raymond et al., 2015; Breig et al., 2016). Not only are they both overexpressed in HCC tissues, where their overexpression was associated with poor prognosis, they both also showed stronger cytoplasmic staining in tumor cells compared to normal hepatocytes (Rousseau et al., 2007; Haurie et al., 2009).

Since their discovery in HCC and colorectal cancer, many other groups reported the involvement of these two ATPases in several cancer types that affect various organs of the body (Grigoletto et al., 2011) (Table 1). This suggested that Pontin and Reptin may play a fundamental role in cancer development, requiring further investigation to consolidate their functions and whether their contribution or regulation of tumor progression is specific to each type of cancer or can be generalized to most.

**Table 1:** Overexpression of Pontin/Reptin in various cancer types.

**System affected**	**Tissue affected**	**Cancer type**	**Abbreviations**	**Pontin**	**Reptin**	**Patient sample**	**Cell line**	**Potential as biomarker examined**	**References**
Digestive	Esophagus	Esophageal squamous cell carcinoma	ESCC	X		X		X	Tung et al., 2013
	Stomach	Gastric cancer		X	X	X	X		Li et al., 2010; Cui et al., 2016
	Pancreas	Pancreatic ductal adenocarcinoma	PDAC	X			X		Taniuchi et al., 2014
	Liver	Hepatocellular carcinoma	HCC	X	X	X	X	X	Rousseau et al., 2007; Haurie et al., 2009; Menard et al., 2010; Tao et al., 2014; Raymond et al., 2015; Breig et al., 2016
	Colon, Rectum	Colorectal cancer	CRC	X	X	X	X		Lauscher et al., 2007, 2012; Milone et al., 2016
Excretory	Kidney	Renal cell carcinoma	RCC	X	X	X	X		Ren et al., 2013; Zhang et al., 2015
	Bladder	Micropapillary carcinoma		X		X			Guo et al., 2016
Respiratory	Lung	Non-small cell lung cancer	NCSLC	X	X	X	X	X	Dehan et al., 2007; Yuan et al., 2016; Velmurugan et al., 2017
	Lung	Small cell lung cancer	SCLC	X	X	X		X	Ocak et al., 2014; Uribarri et al., 2014
Reproductive	Breast	Early-stage breast cancer and Ductal carcinoma *in situ*	DCIS	X		X	X	X	Lacombe et al., 2013, 2014; Su et al., 2014
	Ovary	Ovarian		X		X			Yang et al., 2012
Immune	White blood cell	Acute Myeloid Leukemia	AML	X	X		X		Osaki et al., 2013; Breig et al., 2014
	White blood cell	Lymphoma		X		X	X		Baron et al., 2016

Within the digestive system (Table 1), Pontin and/or Reptin were implicated in cancers of the esophagus, stomach, colon, and pancreas (Li et al., 2010; Lauscher et al., 2012; Tung et al., 2013; Taniuchi et al., 2014; Cui et al., 2016). Specifically, Pontin was implicated in the survival and proliferation of gastric cancer cells and in promoting the invasiveness and migration of pancreatic ductal adenocarcinoma (PDAC) cells (Taniuchi et al., 2014; Cui et al., 2016). Pontin overexpression was correlated with adverse response to adjuvant therapy in colorectal cancer and with poor prognosis for advanced tumor stages. It was found that Pontin levels can be used as a biomarker to discriminate esophageal squamous-cell carcinoma (ESCC) from normal tissue (Lauscher et al., 2007, 2012; Tung et al., 2013). On the other hand, Reptin was shown to be overexpressed in primary tissue of gastric and colon cancers. Reptin overexpression was correlated with aggressive colorectal cancer in a cell model (Li et al., 2010; Flavin et al., 2011; Milone et al., 2016).

In the excretory system (Table 1), overexpression of the two ATPases was found in renal cell carcinoma (RCC) (Ren et al., 2013; Zhang et al., 2015). Like in HCC patients, cytoplasmic localization of Pontin and Reptin in RCC was found to be correlated with metastasis and unfavorable outcome (Rousseau et al., 2007; Haurie et al., 2009; Ren et al., 2013; Zhang et al., 2015). Whether correlation with localization of the protein can apply to other cancer types where cytoplasmic expression was also shown remains to be investigated. Along the same vein, Pontin was found to be overexpressed in the more aggressive and metastatic form of bladder cancer, micropapillary carcinoma (Guo et al., 2016).

Several studies have reported Pontin and/or Reptin expression in both non-small cell lung cancer (NLSCLC) and small cell lung cancer (SCLC) and suggested their potential use as biomarkers for diagnosis and prognosis of lung cancer (Dehan et al., 2007; Ocak et al., 2014; Uribarri et al., 2014; Yuan et al., 2016; Velmurugan et al., 2017) (Table 1).

Pontin was also identified in screens of biomarker/autoantigen panels for ductal carcinoma *in situ* (DCIS) as well as node negative early stage breast cancers (Table 1) (Lacombe et al., 2013, 2014). This could prove to be important for early diagnosis of DCIS and could be a complement to mammography. Functionally, Pontin and Reptin were found to be important in breast cancer cell models in the context of elevated snoRNA and hypertrophy of the nucleolus (Su et al., 2014).

Lastly, these two proteins were shown to be important in cancers of white blood cells, resulting in lymphomas and leukemia (Table 1). Specifically, BCL6, a transcriptional repressor essential for B and T cell development and differentiation, repressed Pontin expression in lymphoma cells (Baron et al., 2016). In addition, Pontin and Reptin were critical regulators of AML1-ETO (in acute myeloid leukemia) and MLL-AF9 (in mixed lineage leukemia), respectively, where their ATPase activities were required for clonogenesis and survival of the cancer cells (Osaki et al., 2013; Breig et al., 2014).

## Role of pontin/reptin in specific cellular pathways

Recent work on Pontin/Reptin attempted to uncover their roles in cellular pathways and processes leading to tumor development. Here, we will discuss the role of these proteins in seven main processes: (1) assembly of replication machinery, (2) aggresome formation, (3) regulation of cell cycle checkpoint, (4) proper mitotic progression, (5) transcriptional regulation, (6) DNA damage response, and (7) cell invasion/migration.

### Assembly of replication machineries by the R2TP complex

**Figure 1:**
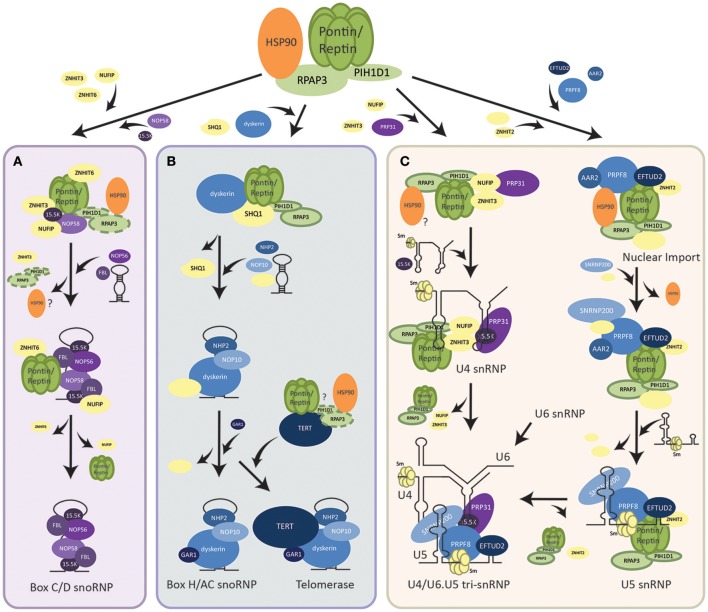
Assembly pathways of RNP complexes regulated by R2TP. **(A)** Assembly of box C/D snoRNP. R2TP facilitates the pre-assembly of box C/D snoRNP components (shown in purple) along with other assembly factors (shown in yellow). PIH1D1 and RPAP3 may dissociate from this pre-snoRNP complex earlier than Pontin/Reptin as other snoRNP proteins and the snoRNA are brought to interact. Pontin/Reptin along with ZNHIT6 and NUFIP dissociate last and mature box C/D snoRNP is translocated into the nucleolus where it functions. **(B)** Assembly of box H/ACA snoRNP and the telomerase holoenzyme. R2TP facilitates the dissociation of SHQ1 assembly factor from box H/ACA snoRNP protein dyskerin. Other snoRNP core proteins (shown in blue), assembly factors (shown in yellow), as well as, the snoRNA are then assembled with the free dyskerin. TERT, the catalytic subunit of the telomerase, may also be bound by the R2TP complex for its assembly with the rest of the snoRNP. **(C)** Assembly of U4 and U5 snRNPs. For U4 snRNP, R2TP along with co-factors (shown in yellow) pre-assembles with PRP31 (shown in purple). Recruitment of 15.5 K then promotes binding of the U4 snRNA. For U5 snRNP, an intermediate complex is first assembled in the cytoplasm by R2TP and HSP90. After nuclear import, the snRNA, and other snRNP proteins are incorporated. U4 can then form a tri-snRNP with U5 and U6.

Pontin and Reptin are established critical regulators of cell growth and proliferation. One of the ways they achieve this is through the assembly of multiple molecular complexes belonging to the replication machinery, largely mediated by the HSP90-interacting chaperone-like complex R2TP (Boulon et al., 2012; Von Morgen et al., 2015), which was discovered by our group (Zhao et al., 2005). R2TP consists of four proteins and is conserved from yeast to humans (Nano and Houry, 2013). Pontin and Reptin are two of the components of the complex, and they interact with PIH1D1 and RPAP3 to form R2TP. Whereas, RPAP3 can bind HSP90 through its TPR domain, PIH1D1 has been proposed to act as an adaptor for the complex and targets R2TP to its clients such as NOP58 of box C/D snoRNP in yeast, dyskerin core factor of box H/ACA snoRNP in mammalian cells, RPB1 subunit of RNA polymerase II in yeast and mammalian cells, and Tel2 of the TTT complex that interacts with mTOR in yeast and mammalian cells (Boulon et al., 2010; Machado-Pinilla et al., 2012; Kim et al., 2013; Kakihara et al., 2014) (Figure 1).

#### Role of pontin/reptin in RNP biogenesis

First found to be important for the biogenesis of box C/D small nucleolar RNP (snoRNP), the role of R2TP has now expanded to the assembly of RNPs of the L7Ae family members (Boulon et al., 2008; McKeegan et al., 2009). In addition to box C/D snoRNPs, this family also consists of box H/ACA snoRNPs (including telomerase), U4 small nuclear RNPs (snRNPs), and selenoprotein mRNAs (Boulon et al., 2008; Machado-Pinilla et al., 2012; Bizarro et al., 2014, 2015). Generally, snoRNPs consist of a small RNA bound by a conserved set of four proteins (Watkins and Bohnsack, 2012). They catalyze specific post-transcriptional modifications on premature rRNAs that are essential for the biogenesis/function of the ribosome: box C/D snoRNPs act in 2′-O-methylation, while box H/ACA snoRNPs guide pseudouridylation of pre-rRNAs (Lui and Lowe, 2013).

##### Assembly of box C/D snoRNPs

Recently, overexpression of snoRNAs has been implicated in the tumorigenesis of several cancers, such as small-cell lung cancer, prostate cancer, breast cancer, and neuronal tumors (Mei et al., 2012; Williams and Farzaneh, 2012; Su et al., 2014; Herter et al., 2015). Elevated snoRNAs support ribosome biogenesis, nucleolar hypertrophy (a common feature in cancer), and protein synthesis for the proliferation of cancer cells (Ruggero and Pandolfi, 2003; Montanaro et al., 2008). In addition, snoRNPs are established oncogene MYC targets, and elevated snoRNP component Fibrillarin was recently found to inactivate tumor suppressor p53 in a cap-independent mechanism (Su et al., 2014; Herter et al., 2015). Thus, regulation of the assembly and biogenesis of snoRNPs (reviewed in Massenet et al., 2016) would also be critical for tumorigenicity.

Though many models have been proposed for the nuclear biogenesis of the box C/D snoRNPs in both yeast and humans, the specific mechanisms and sequence of assembly steps of its core proteins (Fibrillarin, NOP56, NOP58, and 15.5K) by the array of biogenesis factors is still under debate. One hypothesis is that the PIH1D1 and RPAP3 of R2TP act as loading factors for Pontin and Reptin onto core snoRNP proteins NOP58 and 15.5K. Subsequently, PIH1D1 and RPAP3 dissociate from this complex (Bizarro et al., 2014) (Figure 1A). Pontin/Reptin alone with other assembly factors, NUFIP, ZNHIT3, and ZNHIT6 form a pre-snoRNP complex that can be stable independent of RNA (Bizarro et al., 2014; Verheggen et al., 2015). As additional core snoRNP proteins and snoRNA are brought in, the assembly factors are replaced. Pontin/Reptin as well as NUFIP are the last to dissociate from the mature box C/D snoRNP (Bizarro et al., 2014) (Figure 1A). This is supported by evidence that Pontin and Reptin bound differentially to snoRNP proteins and PIH1D1 in an ATP-dependent manner (McKeegan et al., 2009; Cheung et al., 2010b). Whereas, snoRNP 15.5K interacted with Pontin/Reptin when loaded with ATP, the addition of ATP *in vitro* has been shown to dissociate PIH1D1 and RPAP3 from R2TP (McKeegan et al., 2007, 2009). In addition, pulldown assays using snoRNP core proteins as bait were unable to find PIH1D1 nor RPAP3 as interactors (Bizarro et al., 2014).

Another hypothesis is that R2TP as a complex, along with other assembly factors interact and stabilize Nop58 to allow its assembly on the snoRNA with other core snoRNP proteins (Kakihara and Saeki, 2014; Kakihara et al., 2014). This hypothesis is supported by the observations that PIH1D1 interacts *in vitro* with multiple snoRNP proteins such as NOP58, NOP56, and Fibrillarin, and that PIH1D1 is able to immunoprecipitate endogenous or transfected snoRNA (Watkins et al., 2004; McKeegan et al., 2007, 2009; Boulon et al., 2008; Prieto et al., 2015). R2TP proteins were also seen to interact with snoRNP proteins along with other assembly factors such as NUFIP and ZNHIT6, further supporting this hypothesis (McKeegan et al., 2007; Boulon et al., 2008). Further research is needed to elucidate the step-wise assembly of the box C/D snoRNP. Regardless, Pontin and Reptin are essential assembly factors of snoRNP biogenesis, shown to bridge interactions between multiple core proteins.

##### Assembly of box H/ACA snoRNPs

The other major class of snoRNPs are box H/ACA consisting of a snoRNA with a box H/ACA sequence motif that guides the complex to its rRNA target and of four conserved proteins: dyskerin, GAR1, NOP10, and NHP2 (Mannoor et al., 2012). R2TP was found to be essential for the assembly of this snoRNP as well (Figure 1B). Core protein dyskerin is normally bound by assembly factor SHQ1 and prevented from forming the mature snoRNP (Machado-Pinilla et al., 2012). All components of the R2TP complex were required for the dissociation of SHQ1 from dyskerin, though only Pontin/Reptin and PIH1D1 interacted directly with dyskerin (Machado-Pinilla et al., 2012). Pontin/Reptin also directly interacted with SHQ1. This suggested a model where PIH1D1 targeted Pontin and Reptin to the dyskerin-SHQ1 complex, thereby allowing Pontin and Reptin to remove SHQ1 from dyskerin (Machado-Pinilla et al., 2012) (Figure 1B). Whether this process is through competitive binding or dependent on the ATPase activity of Pontin and Reptin to induce conformational changes in the dyskerin-SHQ1 complex is uncertain. The role of RPAP3 in this process is also not clear.

##### Assembly of the telomerase complex

The human telomerase complex is composed of the telomerase reverse transcriptase enzyme TERT and the TERC RNP consisting of the telomerase RNA component TERC (which contains a box H/ACA motif) along with all four proteins of the box H/ACA snoRNP family (Maciejowski and de Lange, 2017). Thus, it can also be considered as being a member of the box H/ACA class. Pontin and Reptin were found to play a critical role in the assembly and activity of telomerase through interacting with both TERT and the TERC RNP (Venteicher et al., 2008). Thus, their role in TERC RNP assembly may follow that of the canonical box H/ACA snoRNP, where R2TP dissociates dyskerin from SHQ1, allowing the free dyskerin to interact and associate with other snoRNP proteins (Figure 1B).

Telomerase is responsible for adding telomere repeats to chromosome ends, protecting them from DNA damage or erosion (Maciejowski and de Lange, 2017). In differentiated human somatic cells, TERT is silenced and telomeres undergo programmed shortening, eventually leading to cell growth arrest as well as senescence or apoptosis. However, telomerase is upregulated in cancer, enabling indefinite proliferation of the cells and the development of tumors (Li and Tergaonkar, 2014; Maciejowski and de Lange, 2017). Pontin/Reptin can regulate TERT both on the gene and protein levels (Venteicher et al., 2008; Li et al., 2010; Flavin et al., 2011). Though both Pontin and Reptin were needed for the accumulation of TERT mRNA, only Reptin depletion inhibited TERT promoter activity; this is likely through the regulation of MYC (c-myc), the transcription factor for TERT (Li et al., 2010). Reptin was found to bind MYC at the promoter region of TERT, and when Reptin was depleted, MYC was unable to bind to the E-box motif (the MYC-binding motif) on the TERT promoter (Venteicher et al., 2008; Li et al., 2010). Thus, it is intriguing to hypothesize that a silencing factor/repressor may usually bind this region, and that Reptin assists MYC in displacing the repressor thus allowing transcription of TERT.

Venteicher et al. (2008) found that Pontin directly interacts with the TERT protein in complex with Reptin, forming a TERT-Pontin/Reptin complex. However, the enzymatic activity of TERT in this complex is significantly lower than that when TERT is associated with the TERC RNP member dyskerin. During the cell cycle, the interaction between TERT and the ATPases peaks in S phase and diminishes in G2, M, and G1. This suggests that Pontin and Reptin may be binding to a pre-telomerase TERT that needs remodeling or association with other factors for its activity (Venteicher et al., 2008). One hypothesis is that Pontin and Reptin may act again as assembly factors as part of the R2TP complex and dissociate after the mature telomerase complex is formed (Venteicher et al., 2008; Machado-Pinilla et al., 2012). This is supported by observations that HSP90 functions in the nuclear import of TERT (Lee and Chung, 2010; Jeong et al., 2016). It may also be possible that Pontin and Reptin hold TERT in an inactive form until TERT activity is needed.

##### Assembly of spliceosomal snRNP U4 and U5

The spliceosome is comprised of five snRNPs (U1, U2, U4, U5, and U6) that cooperatively mediate the splicing of pre-mRNAs for proper gene expression (Matera and Wang, 2014). U4, U5, and U6 are recruited to the splicing site as a tri-snRNP complex and then rearranged into a catalytically active complex (Nguyen et al., 2015). In addition to the snRNA, each snRNP contains a heptameric ring of either Sm or Like-Sm proteins, as well as a variable number of snRNP-specific proteins (Matera and Wang, 2014). The assembly of snRNP-specific proteins has recently been proposed to be regulated by the R2TP complex along with HSP90 (Bizarro et al., 2015; Cloutier et al., 2017; Malinova et al., 2017).

The assembly of snRNPs generally begins with the export of snRNAs out of the nucleus (Matera and Wang, 2014). In the cytoplasm, the Sm ring is loaded onto the snRNA by the SMN complex and reimported (Battle et al., 2006). Assembly of U4-specific proteins PRP31 and 15.5K into the snRNP by R2TP, NUFIP and ZNHIT3 is thought to occur after reimport into the nucleus (Figure 1C) (Bizarro et al., 2015). PRP31 first forms a complex with R2TP and assembly factors, then binding of 15.5K promotes the stable incorporation of PRP31 into the snRNP (Bizarro et al., 2015).

On the other hand, assembly of the U5-specific proteins occurs first in the cytoplasm (Figure 1C) (Malinova et al., 2017). An intermediate complex is formed with the recruitment of PRPF8 and EFTUD2 to the R2TP/HSP90 complex along with AAR2, ZNHIT2, and other assembly factors (Cloutier et al., 2017). HSP90 is thought to stabilize PRPF8 and EFTUD2 through the interaction of PIH1D1 N-terminal domain with the phosphorylated DSDED motif on EFTUD2 (Malinova et al., 2017). After the nuclear import of this complex, binding of SNRNP200 and other cofactors occurs, followed by binding of U5 snRNA and release of assembly factors for the maturation of U5 snRNP (Malinova et al., 2017) (Figure 1C). Finally, U4, U5, and U6 snRNPs assemble together to form the U4/U6.U5 tri-snRNP (Figure 1C).

#### Assembly of RNA polymerase II

**Figure 2:**
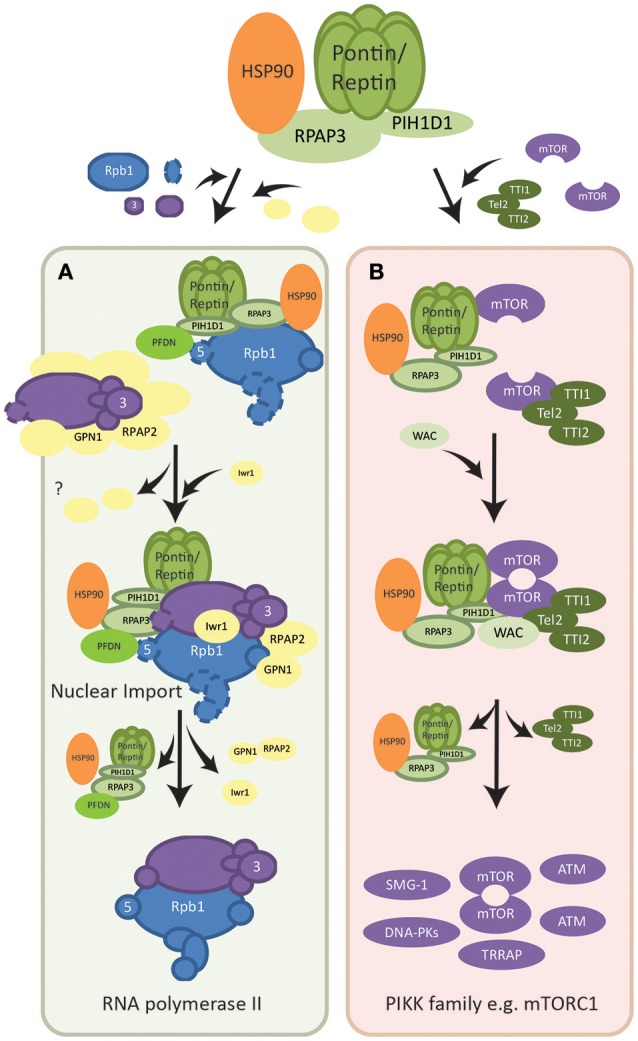
Assembly pathways of macromolecular complexes regulated by R2TP. **(A)** Assembly of RNA polymerase II. Two subcomplexes of the polymerase, RPB1-associated (shown in blue) and RPB3 associated (shown in purple), are formed with the help of R2TP/PFDN and other assembly factors (shown in yellow). Assembly factors dissociate as the mature RNA polymerase II is formed and Iwr1 importin is brought in. Fully assembled RNA polymerase II is then translocated into the nucleus also mediated by assembly factor RPAP2. The point at which R2TP/PFDN dissociate is not known. RPAP2 dissociates in the nucleus and is recycled by being co-exported with GPN1. **(B)** Dimerization of mTOR complex. Each mTOR subunit is bound by either the TTT complex (dark green) or the R2TP complex (light green). The WAC adaptor facilitates the interaction between these two complexes for the dimerization of mTOR. The assembly factors then dissociate from the dimerized and activated mTOR complex. R2TP is also involved in the assembly/stability of other PIKK family members (shown in purple), however the molecular basis of its functions is poorly understood.

RNA polymerase II (POL II) is a fundamental cellular complex that synthesizes all the mRNAs and capped non-coding RNAs. Its 12 subunits are assembled in the cytoplasm, in part by the R2TP complex, and only fully assembled POL II is imported into the nucleus (Boulon et al., 2010). The subunits are formed in two subcomplexes: RPB1-associated and RPB3-associated complexes, each interacting with a specific set of assembly factors (Boulon et al., 2010, 2012) (Figure 2A). R2TP along with a set of six proteins that form a prefoldin-like (PFDN-like) complex (PFD2, PFD6, PDRG1, UXT, URI, and WDR92) named the R2TP/PFDN complex (Boulon et al., 2010; Millan-Zambrano and Chavez, 2014), was found to interact with the POL II RPB1 subcomplex (Figure 2A). Free RPB1 subunits in the cytoplasm were mainly stabilized by HSP90 via interactions with RPAP3, facilitating the association and assembly of RPB1 with other subunits (Boulon et al., 2010). In addition, URI of the R2TP/PFDN complex interacted with RPB5, another subunit of the RPB1 subcomplex, further implicating R2TP/PFDN in the assembly of POL II (Mita et al., 2013). Fully assembled POL II is then transported into the nucleus via the Iwr1 import adaptor, as well as assembly factor RPAP2 and GTPase GPN1/RPAP4 (Boulon et al., 2010; Forget et al., 2013). While RPAP2 mediated the nuclear import of POL II, GPN1/RPAP4 is required for the recycling of RPAP2 by exporting it back into the cytoplasm in a CRM1-dependent manner (Forget et al., 2013).

Intriguingly, RPAP3 was found to interact with subunits of both RNA polymerase I and III (Jeronimo et al., 2007; Boulon et al., 2010). In addition, the URI interactor RPB5 is a subunit common to all three RNA polymerases, suggesting that the R2TP/PFDN complex may function in the assembly of RNA polymerases in general (Mita et al., 2013). If so, this may partly explain the overexpression of Pontin and Reptin in many cancers, as their supporting role in protein synthesis and gene expression will help meet the high demand in proliferating tumor cells.

#### Assembly of mTORC1 and other PIKK family members

PIKK (phosphatidylinositol 3-kinase-related protein kinase) signaling family important for DNA repair and cellular metabolism (Bakkenist and Kastan, 2004) comprises of six members including mTOR (mechanistic target of rapamycin), SMG-1 (suppressor with morphogenetic effect on genitalia-1), ATM (ataxia telangiectasia mutated), ATR (telangiectasia Rad3-related), DNA-PKcs (DNA-dependent protein kinase catalytic subunit), and TRAAP (transformation/transcription domain-associated protein) (Baretic and Williams, 2014). Pontin and Reptin regulate mTOR as well as other members of the PIKK signaling family at the transcriptional level, protein level, and functionally (Dugan et al., 2002; Horejsi et al., 2010; Izumi et al., 2010; Kim et al., 2013). This was shown by the decrease in both mRNA and protein levels of PIKK members upon depletion of either Pontin or Reptin (Izumi et al., 2010), and, consequently, downstream signaling was also affected. Evidence suggested that Pontin and Reptin regulate transcription factors such as E2F1, whose target genes include members of the PIKK family (Dugan et al., 2002; Taubert et al., 2004; Tarangelo et al., 2015).

Interactors of R2TP, such as TEL2 of the TTT complex (Tel2, Tti1, and Tti2; Figure 2B) were shown to be essential for the protein stability of all members of the PIKK family (Takai et al., 2007; Horejsi et al., 2010, 2014; Pal et al., 2014). This interaction is mediated by the phosphoserine-containing motif DpSDD/E on TEL2 interacting with the N-terminal domain of PIH1D1 (Horejsi et al., 2010; Pal et al., 2014). In addition, the HSP90 chaperone was found to be required for the accumulation of PIKK proteins, likely through its cofactor RPAP3 (Izumi et al., 2010; Pal et al., 2014). Pontin and Reptin, perhaps through the R2TP complex, were shown to be directly involved in the remodeling and assembly of complexes formed by PIKK members. For instance, the ATPases promoted the remodeling of mRNA surveillance complexes, of which SMG-1 is a subunit, during nonsense-mediated mRNA decay (Izumi et al., 2010).

In addition, Pontin and Reptin were shown to be important for the localization and dimerization/activation of the mTORC1 complex under metabolic stress (Kim et al., 2013; David-Morrison et al., 2016). mTOR is a serine/threonine kinase that senses cellular nutrients and energy levels to regulate metabolism and physiology in mammalian cells. It is the catalytic subunit of two distinct complexes named mTORC1, which controls cell growth and protein synthesis, and mTORC2, responsible for cell survival signaling. PIH1D1 was also shown to be important for the assembly of mTORC1 complex components (Kamano et al., 2013). A recent model suggested that Pontin/Reptin associated with TTT to form a Pontin/Reptin-TTT complex under energy-rich conditions, helped by the adaptor WAC (David-Morrison et al., 2016). This complex then facilitated the dimerization and proper localization of mTORC1 to the lysosome in an energy-dependent manner (Kim et al., 2013; David-Morrison et al., 2016) (Figure 2B).

Functionally, R2TP has been shown to promote mTORC1-dependent transcription of rRNA, and thus ribosome biogenesis (Kamano et al., 2013). The R2TP-mTORC1 interaction is thought to be mediated by PIH1D1, which only interacted with mTORC1 complex components but not mTORC2 (Kamano et al., 2013; Horejsi et al., 2014) (Figure 2B).

Taken together, Pontin and Reptin can regulate the function of many macromolecular complexes within the cell, sometimes on multiple different levels throughout a pathway. It is therefore expected that defects in any of these pathways can have considerable impact on cell growth.

### Role of pontin/reptin in aggresome formation

Aggresome formation is a highly-regulated process that protects the cell from aggregating polypeptides when its protein degradation and chaperone systems are overwhelmed. Aggresomes are formed from aggregated and misfolded polypeptides that are transported to a centralized location near/around the centrosomes (Johnston et al., 1998; Markossian and Kurganov, 2004).

Pontin and Reptin were identified in a siRNA screen for proteins involved in aggresome formation (Zaarur et al., 2015). Depletion of the two ATPases led to the build-up of scattered cytoplasmic aggregates and reduced the formation of centralized aggresomes. It was found that Pontin and Reptin interacted and co-localized with synphilin-1, previously shown to accumulate in and form cytoprotective aggresomes (Tanaka et al., 2004; Zaarur et al., 2015). Additionally, Pontin/Reptin were found to promote disassembly of protein aggregates *in vivo*. Thus, Pontin and Reptin may function as disaggregating chaperones, and/or be indirectly involved in aggresome formation.

### Role of pontin/reptin in cell cycle regulation

**Figure 3:**
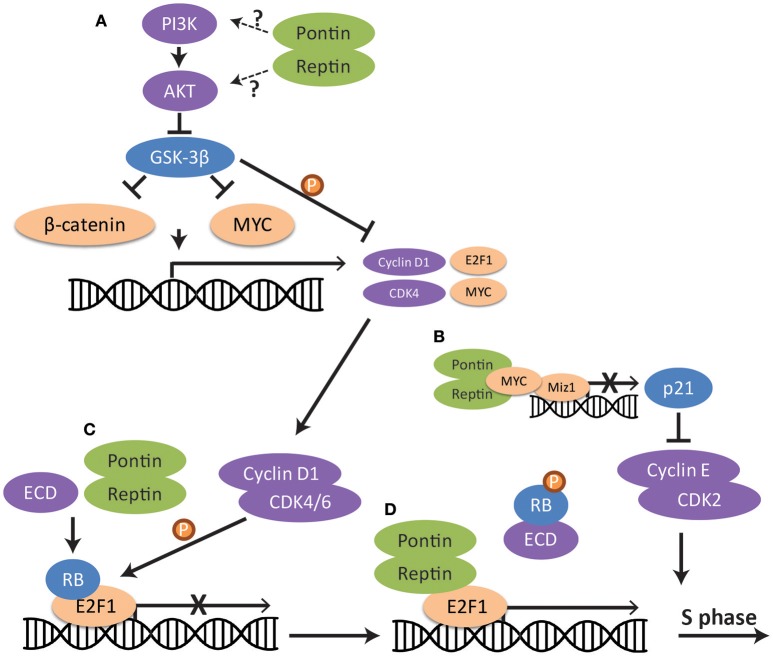
Regulation of the G1/S cell cycle checkpoint pathway by Pontin/Reptin at multiple levels. **(A)** PI3K-AKT-GSK-3β signaling. Pontin/Reptin activate the PI3K/AKT pathway upstream of GSK-3β, leading to the inhibition of GSK-3β and release of the repression of GSK-3β on β-catenin, MYC and cyclin D1. **(B)** MYC repression of p21 transcription. Pontin promotes interaction between MYC and MIZ1 at the p21 promoter. This represses the transcription of p21 and allows cyclin E and CDK2 to promote S-phase entry. **(C)** RB dissociation from E2F1 transcription factor through ECD. Pontin promotes ECD-mediated dissociation of Retinoblastoma protein from E2F1. **(D)** E2F1 transcription of cell cycle genes. Pontin and Reptin in complex with TIP60 promote E2F1 transcription of cell cycle genes during late G1 phase.

Studies in a variety of cancer cell lines have consistently demonstrated that downregulation of either Pontin or Reptin may lead to cell cycle arrest at the G1/S phase checkpoint, resulting in the accumulation of cells in G1 and a reduction of cells in all other phases of the cell cycle (S, G2/M) (Rousseau et al., 2007; Haurie et al., 2009; Menard et al., 2010; Osaki et al., 2013; Ren et al., 2013; Breig et al., 2014; Zhang et al., 2015; Yuan et al., 2016). G1/S transition is regulated by many proteins and pathways that are normally inactivated until entry into S phase is signaled (Otto and Sicinski, 2017). For example, E2F1 transcription factor, responsible for the expression of a collection of S-phase promoting genes, is normally held in the inactive state by retinoblastoma proteins (RB) (Johnson et al., 2016) (Figure 3). This E2F1-RB complex is phosphorylated by cyclin D1 and, consequently, E2F1 dissociates from RB and becomes able to act on its target genes (Malumbres and Barbacid, 2001). However, cyclin D1 and other cell cycle genes are only upregulated when mitogenic signals activate downstream pathways such as the PI3K/AKT signaling pathway (Hustedt and Durocher, 2016). This in turn activates transcription factors such as MYC and β-catenin for the expression of cell cycle proteins, including cyclin D1 (Shtutman et al., 1999; Liao et al., 2007). GSK-3β is an important inhibitor of MYC and β-catenin as well as of cyclin D1 when cells are not ready for entry into S phase (Domoto et al., 2016). However, active AKT phosphorylates and inhibits GSK-3β, releasing its repression (McCubrey et al., 2016) (Figure 3).

Research has shown the importance of Pontin and Reptin in various steps of the G1/S cell cycle checkpoint pathway (Figure 3). Silencing of Pontin in lung adenocarcinoma led to the phosphorylation and degradation of cyclin D1, thus resulting in cell cycle arrest at G1/S (Yuan et al., 2016). Evidence suggested that Pontin acts upstream of GSK-3β through the AKT/GSK-3β/cyclin D1 pathway (Figure 3A), though how Pontin functions in the activation of this signaling pathway remains to be elucidated.

Additionally, Pontin and Reptin were shown as interactors of MYC and β-catenin (see section Role of Pontin/Reptin in Mitosis for details), and thus can potentially regulate their transcriptional activity for production of cyclins (Bauer et al., 2000; Wood et al., 2000). In RCC cells, Pontin knockdown led to a decreased mRNA expression of both MYC and cyclin D1 (Zhang et al., 2015). Pontin and Reptin can also regulate the ability of MYC to enhance cell-cycle progression by stimulating its inhibition of the transcription factor MIZ1 (Etard et al., 2005). Consequently, its target p21, which inhibits cyclin protein activity, is transcriptionally repressed (Figure 3B) (Etard et al., 2005; Hustedt and Durocher, 2016).

Further downstream in the signaling pathway, Pontin may be needed for the dissociation of RB from E2F1 through the interaction with ecdysoneless (ECD) (Figure 3C). ECD is an evolutionarily conserved protein essential for embryogenesis and cell cycle progression into S phase (Kim et al., 2009). It competes with E2F1 for binding to RB, thus allowing E2F1 to freely activate its target genes. Pontin may facilitate efficient binding of ECD to RB and dissociation of RB from E2F1, as interaction with Pontin is required for ECD's ability to regulate progression of cell cycle (Mir et al., 2015). Since ECD also contains a DSDD motif and is shown to interact with PIH1D1, Pontin may function as part of the R2TP complex in this process (Horejsi et al., 2014). However, the interaction between PIH1D1 and ECD was shown not to be important for its cell cycle functions (Mir et al., 2015). It is also possible that Pontin and Reptin can promote E2F1 transcription in this context as part of the TIP60 histone acetyltransferase complex, since this complex was seen to be recruited by E2F1 in late G1 phase (Figure 3D) (Taubert et al., 2004).

### Role of pontin/reptin in mitosis

**Figure 4:**
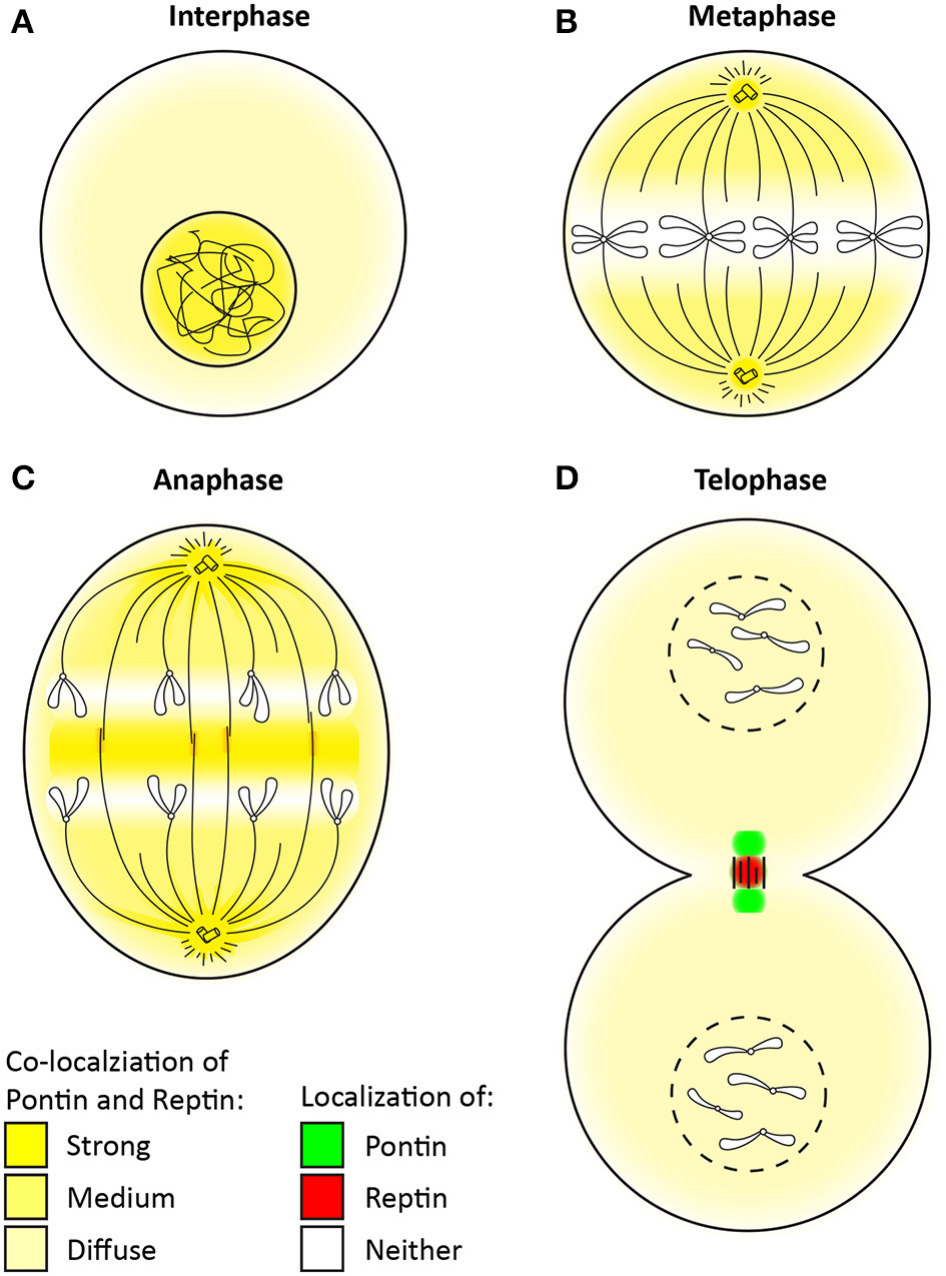
Change in the localization of Pontin/Reptin throughout mitosis. Localization of Pontin/Reptin shown in different colors during: **(A)** Interphase, **(B)** Metaphase, **(C)** Anaphase, and **(D)** Telophase. Chromatin, chromosomes, microtubules, and centrosomes are schematically represented in black lines.

Pontin and Reptin may also play specific and perhaps essential roles in mitosis independent of each other. Whereas, Pontin was largely implicated in the assembly of mitotic spindles, the function of Reptin remains to be uncovered (Gartner et al., 2003; Sigala et al., 2005; Ducat et al., 2008; Fielding et al., 2008; Gentili et al., 2015). However, both Pontin and Reptin undergo dramatic subcellular relocalization during mitosis and even displaying distinct localization signals within the intercellular bridge (Figure 4) (Sigala et al., 2005; Gentili et al., 2015).

During interphase, Pontin/Reptin are mostly nuclear (Figure 4A) (Gartner et al., 2003; Sigala et al., 2005). Upon entry into mitosis, Pontin/Reptin are increasingly redistributed to the cytoplasm, culminating in metaphase, where they are almost completely excluded from the condensed chromosomes (Figure 4B). Here, and until early anaphase, Pontin/Reptin are observed at mitotic spindles and centrosomes, co-localizing with both α- and γ-tubulin (Figure 4C) (Gartner et al., 2003; Sigala et al., 2005; Ducat et al., 2008). During anaphase-to-telophase transition, both relocate to the central spindle, first forming a compact band, then accumulating into distinct foci. At telophase, Pontin was found to form two foci that co-localized with β-tubulin at the sides of the cytokinetic furrow (Figure 4D). On the other hand, Reptin was found to form only one focus that was concentrated at the center of the midbody, separated from Pontin (Gentili et al., 2015).

Both Pontin and Reptin have been found to be part of the microtubule interactome, and were identified as candidate mitotic regulators in a RNAi-based phenotypic screen in Drosophila S2 cells (Bjorklund et al., 2006; Ducat et al., 2008). Depletion of Pontin led to multiple mitotic defects in a variety of mammalian cells, including increased mitotic death, delayed anaphase onset, defective spindles, leading to misaligned and lagging chromosomes (Gartner et al., 2003; Ducat et al., 2008; Magalska et al., 2014; Gentili et al., 2015). Depletion of Reptin on the other hand had little effect by itself, and only enhanced the defects observed with Pontin depletion (Ducat et al., 2008). This suggested that Pontin is the main protein involved in promoting mitotic spindle assembly, likely through regulating the localization of the γ-tubulin ring complex (γ-TuRC) and Integrin linked kinase (ILK) to the mitotic spindle and centrosome (Gartner et al., 2003; Fielding et al., 2008).

γ-TuRC serves as the cap and initiation site for microtubule polymerization (Prosser and Pelletier, 2017). Both Pontin and Reptin were shown to interact with γ-TuRC and were required for the nucleation and organization of robust microtubule arrays in Xenopus egg extracts (Ducat et al., 2008). Perhaps Pontin and Reptin act as chaperones for the stability and localization of γ-TuRC to the spindle poles and along the microtubule array. ILK was recently found to be important in the centrosome for mitotic spindle organization likely by maintaining the interaction between spindle organization proteins Aurora A and TACC3/ch-TOG, in a manner that is dependent on its kinase activity (Fielding et al., 2008). ch-TOG is required for spindle organization and microtubule polymerization, and Aurora A kinase recruits ch-TOG through phosphorylating TACC3. Consequently, depleting ILK led to spindle defects. Pontin and ILK co-localize in the centrosome and were dependent on each other for their localization (Fielding et al., 2008). Thus, they may form a co-complex during mitosis to function in the centrosome (Dobreva et al., 2008).

As mentioned above, later in mitosis, Pontin and Reptin re-localized to the central spindle and even seemed to separate from each other at the midbody (Sigala et al., 2005; Ducat et al., 2008; Gentili et al., 2015). This dissociation is likely regulated by Polo-like kinase 1 (PLK1), a mitotic kinase, that is found to interact and co-localize with Pontin during cytokinesis (Gentili et al., 2015). PLK1 has many critical functions in mitosis, including proper mitotic entry, spindle assembly, centrosome maturation, and chromosome segregation (Petronczki et al., 2008; Otto and Sicinski, 2017). During cytokinesis, PLK1 is required for midbody formation and function, of which Pontin might be a mediator, as Pontin was also found to be a PLK1 substrate *in vitro* (Gentili et al., 2015). However, the specific functions and molecular mechanisms of Pontin and Reptin at the midbody remain to be characterized.

Pontin and Reptin have also been implicated at the end of mitosis in chromatin decondensation (Magalska et al., 2014). Here, they re-associate and were shown to exist largely as a heterocomplex again, although they are functionally redundant in this context and can act independent of one another (Magalska et al., 2014). ATPase-deficient mutants of either protein showed a dominant-negative effect on chromatin decondensation.

### Role of pontin/reptin in the regulation of transcriptional oncogenic factors

Pontin and Reptin have long been recognized to regulate transcription through interaction with different transcription factors, many of which are highly involved in tumorigenesis, including MYC, β-catenin-LEF/TCF, and E2F to name a few (Gallant, 2007; Huber et al., 2008; Grigoletto et al., 2011; Rosenbaum et al., 2013; Matias et al., 2015). The role of Pontin/Reptin in these contexts generally promotes cell proliferation and survival, which is crucial for cancer development (Table 2).

**Table 2:** Regulation of transcription factors by Pontin and Reptin.

**Transcription factor**	**Pontin**	**Reptin**	**Co-factors**	**Target gene**	**Target gene function**	**Regulation**	**References**
MYC	X	X	MIZ1	p21	Cell-cycle inhibitor	Repression	Etard et al., 2005
	X	X	MIZ1	C/EBPδ	Tumour supressor	Repression	Si et al., 2010
	X	X	MIZ1	mfas	Cell adhesion	Repression	Bellosta et al., 2005
	X			rRNA	Ribosomal RNA	Activation	Cvackova et al., 2008; Kamano et al., 2013
		X	ETS2	TERT	Telomerase reverse transcriptase	Activation	Li et al., 2010; Flavin et al., 2011
	X	X	E1A 243R, TIP60 complex	MYC targets	Transformation	Activation	Zhao et al., 2016
E2F1	X	X		Metabolic targets	Glucose metabolism (e.g., Warburg effect)	Activation	Tarangelo et al., 2015
	X	X	TIP60	Cell cycle targets	Cell cycle progression (S-phase entry)	Activation	Taubert et al., 2004
ER (estrogen receptor)	X	X		CCND1	Cyclin D1 (S-phase entry)	Activation	Dalvai et al., 2013
AR (androgen receptor)	X			PSA	Prostate-specific antigen	Activation	Kim et al., 2007
ISGF3 (STAT1, STAT2, IRF9)	X	X		ISG	Interferon α-stimulated genes	Activation	Gnatovskiy et al., 2013
H1F1α	X	X	TIP60	H1F1α targets	Hypoxia signaling	Activation	Perez-Perri et al., 2016
	X			H1F1α targets	Hypoxia signaling	Activation	Lee et al., 2011
		X	HDAC	H1F1α targets	Hypoxia signaling	Repression	Lee et al., 2010
p53	X			Mutp53 targets	Transformation	Activation	Zhao et al., 2015
		X	AGR2	p53 targets	Tumor/proliferation supression	Repression	Maslon et al., 2010; Gray et al., 2013; Clarke et al., 2016
		X	p14^ARF^, MDM2	p53 targets	Tumor/proliferation supression	Repression	Xie et al., 2012
NF-κB		X	IκB-α	NF-κB targets	Inflammation	Repression	Qiu et al., 2015
	X		Bcl3, TIP60	KAI1	Metastasis supressor	Activation	Kim et al., 2005; Rowe et al., 2008
		X	Bcl3, β-catenin, HDAC	KAI1	Metastasis supressor	Repression	Kim et al., 2005, 2006
β-catenin-LEF/TCF (Wnt pathway)	X			Wnt targets	Wnt signaling	Activation	Bauer et al., 1998, 2000
	X		c-FLIP_L_	Wnt targets	Wnt signaling	Activation	Zhang et al., 2017
		X	HDAC	Wnt targets	Wnt signaling	Repression	Bauer et al., 2000
	X	X	Hint1	Wnt targets	Wnt signaling	Repression	Weiske and Huber, 2005
	X	X	APPL1/2	Wnt targets	Wnt signaling	Activation	Rashid et al., 2009
Oct4	X	?	p300	Oct4 targets	ESC maintenance	Activation	Do et al., 2014; Boo et al., 2015
	X	?	p300	lincRNA	Lineage program repression	Activation	Do et al., 2014; Boo et al., 2015

#### The role of pontin/reptin in TIP60 histone acetyl transferase activity

Histone acetylation is an important strategy for the regulation of gene expression as it typically relaxes chromatin structure allowing the binding of the transcriptional machinery to proper promoter regions (Desjarlais and Tummino, 2016). As a histone acetyltransferase, the TIP60 complex acts in a similar fashion and mostly functions as a co-activator of many transcriptional pathways. The TIP60 complex consists of proteins with chromatin remodeling activity such as Pontin/Reptin and p400, adaptor/scaffolding subunits such as TRAAP and DMAP1, histone binding proteins BRD8 and ING3, as well as the histone acetyltransferase TIP60 among others (Desjarlais and Tummino, 2016). The complex is involved in regulating chromatin remodeling, transcription and DNA repair (Kusch et al., 2004; Zhao et al., 2016).

**Figure 5:**
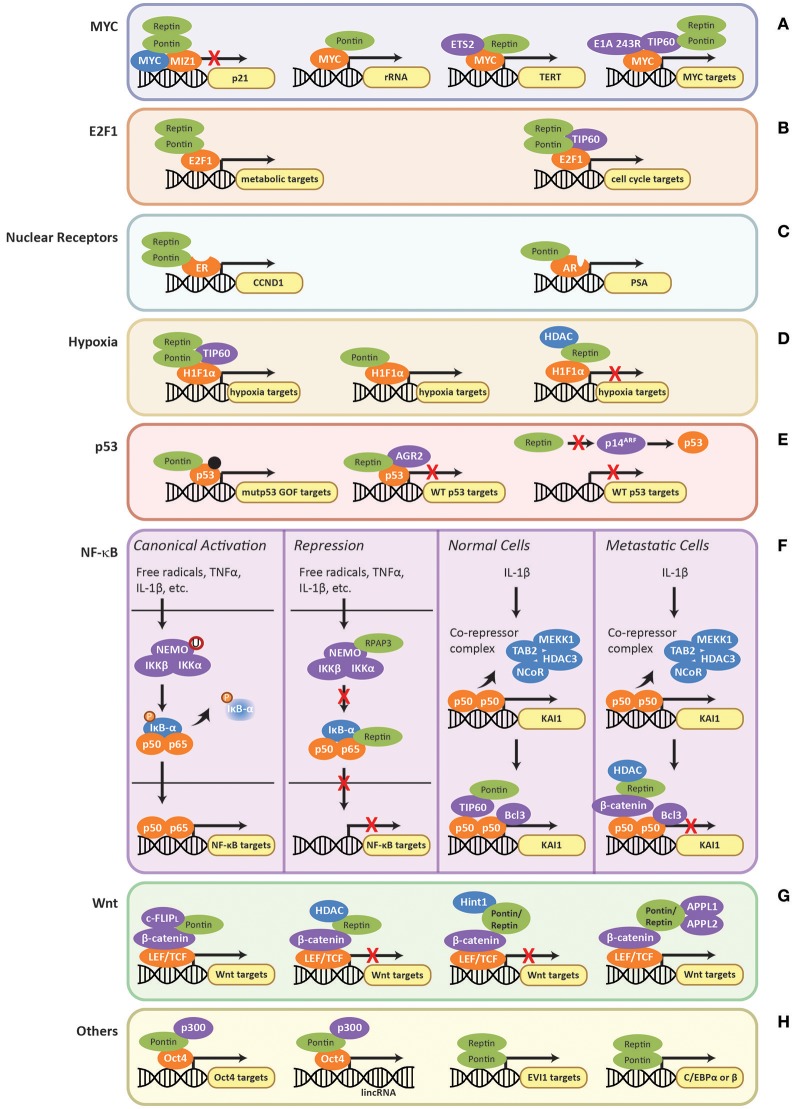
Regulation of transcription factors by Pontin/Reptin. Role of Pontin/Reptin in the regulation of: **(A)** MYC at p21, rRNA, TERT, and other promoters; **(B)** E2F1 at metabolic and cell cycle gene promoters; **(C)** nuclear receptors—estrogen receptor at cyclin D1 (CCND1) promoter and androgen receptor at prostate-specific antigen (PSA) promoter; **(D)** H1Fα at the promoters of different subsets of hypoxia signaling genes; **(E)** Mutant gain-of-function p53, indicated by a black dot, and wild-type p53 at their respective target gene promoters; **(F)** NF-κB target promoters and at KAI1 promoter in normal and metastatic cells; **(G)** LEF/TCF-mediated activation/repression of the Wnt signaling pathway through various co-factors; and **(H)** other transcription factors.

In the context of transcription, the TIP60 complex, or at least components of it, are recruited by several oncogenic transcription factors that are regulated by Pontin and Reptin. For example, the E1A 243R adenoviral oncoprotein was recently found to interact with subunits of the TIP60 complex including Pontin and Reptin as well as MYC (Zhao et al., 2016). E1A 243R promoted the interaction between TIP60 and MYC to form a supercomplex consisting of all three components, which was important for the cellular transformation activities of MYC and E1A (Figure 5A) (Dugan et al., 2002; Zhao et al., 2016). Other transcription factors regulated by Pontin/Reptin also recruit TIP60 including HIF1α and NF-κB, which are further discussed below.

#### Role in MYC regulation

MYC is an oncogenic transcription factor that promotes cell proliferation by transcriptionally activating genes involved in cell cycle progression, protein synthesis, and ribosome biogenesis, including Pontin and Reptin (Dang, 2012). Recently, ChIP-seq analysis showed that MYC binds the promoter regions of both Pontin and Reptin (Walz et al., 2014). MYC also binds to the promoter of genes coding for cell-cycle inhibitors, such as p21 (Etard et al., 2005). MYC is a repressor of p21 through inhibition of the MIZ1 transcription factor (Etard et al., 2005). The direct binding of the two ATPases to MYC oncogenesis domain was shown to be important for MYC/MIZ1 interaction. Here Pontin and Reptin act as co-repressors in an additive manner, thus enhancing the repression of p21 by MYC (Figure 5A) (Wood et al., 2000; Etard et al., 2005).

Pontin and Reptin were also found to be essential for MYC-mediated oncogenic transformation and modulated MYC-induced apoptosis in an ATPase dependent manner (Table 2), where ATPase-deficient mutant of Pontin enhanced apoptosis if MYC was overexpressed (Wood et al., 2000; Dugan et al., 2002). Apoptosis is a common strategy for cells to prevent transformation and uninhibited proliferation. Thus, inhibiting the ATPase activity of Pontin can prove to be therapeutically beneficial. Pontin and Reptin were also shown to be important for the repression of tumor suppressor C/EBPδ and Drosophila cell adhesion gene mfas, both also target genes of MYC, further supporting their roles in MYC-mediated oncogenesis (Bellosta et al., 2005; Si et al., 2010).

On the other hand, Pontin and Reptin can act as activators of MYC-mediated transcription. Within the nucleolus, an interaction of Pontin and MYC at the rRNA promoter was observed (Figure 5A), though the function and the mechanistic aspects of this interaction are still unclear (Cvackova et al., 2008). Recent findings suggested that rRNA transcription might also be regulated by the R2TP complex indirectly through its interactions with mTORC1 (Kamano et al., 2013). Reptin was shown to activate MYC-dependent transcription of TERT (Figure 5A) in cooperation with ETS2, a transcription factor acting downstream of growth factor signaling (Li et al., 2010; Flavin et al., 2011).

It was recently revealed that MTBP (Mdm2-binding protein) may be involved in the interactions between Pontin/Reptin and MYC (Grieb et al., 2014). MTBP associated with MYC at its target promoters through direct binding with Pontin and Reptin (Grieb et al., 2014). Co-overexpression of MYC and MTBP resulted in dramatic increase in proliferation and transformation experimentally, and correlated with a 10-year reduction in patient survival (Grieb et al., 2014). It would be interesting to investigate whether MTBP also co-overexpressed with Pontin and Reptin in patient samples and whether MTBP is involved in regulating Pontin and Reptin interaction with other transcription factors and/or protein complexes.

#### Role in E2F1 regulation

A similar role for Pontin/Reptin in MYC-mediated transformation and oncogenesis was observed for transcription factor E2F1, an important regulator of cell cycle, to which Pontin also directly binds (Dugan et al., 2002). Using a pre-clinical mice model of HCC, Tarangelo et al. (2015) reported that Pontin and Reptin were recruited by transcription factor E2F1 to open the chromatin at E2F1 target genes, which in turn enhanced the transcriptional response of metabolic genes during cancer progression (Figure 5B). Here, Pontin/Reptin act as co-activators for E2F1 (Table 2). However, whereas Reptin ATPase activity was required for chromatin remodeling, the role of Pontin seemed limited to stabilizing Reptin expression (Tarangelo et al., 2015). The authors suggested that the recruitment of Pontin and Reptin may be a common mechanism used by E2F1 to promote cancer progression. Through ChIP-seq studies, the authors also showed that the chromatin remodeling effects of Pontin and Reptin were not through the TIP60 histone acetyltransferase (HAT) complex that Pontin and Reptin are subunits of (as described above), since TIP60 was not observed at the promoters of E2F1 target genes in this model of HCC. However, TIP60 recruitment along with Pontin and Reptin by E2F1 was seen in the context of cell cycle gene transcription (Figure 5B) (Taubert et al., 2004).

Their cooperative action as co-activators was also observed in the context of regulating nuclear receptors, estrogen receptor (ER) and androgen receptor (AR), as well as the transcription factor complex interferon stimulated-gene factor 3 (ISGF3) (Figure 5C and Table 2) (Kim et al., 2007; Dalvai et al., 2013; Gnatovskiy et al., 2013).

#### Role in HIF1α regulation

The TIP60 complex, including both Pontin and Reptin subunits, has recently been found to regulate the hypoxia pathway through co-activating the transcription factor HIF1α (hypoxia-inducible factor alpha) (Perez-Perri et al., 2016). Transcriptome analysis showed that more than 60% of HIF1α target genes utilized either TIP60, CDK8-Mediator, or both as co-activators (Perez-Perri et al., 2016). In cancer, due to uncontrolled proliferation of cells, the tumor and its microenvironment are often deprived of oxygen (Wilson and Hay, 2011). This signals the hypoxic response to alter cellular metabolism for better adaptation (Perez-Perri et al., 2016). However, this often leads to angiogenesis, epithelia-to-mesenchymal transition (EMT), metastasis, apoptosis, and resistance to treatments (Wilson and Hay, 2011). Thus, understanding and modulating hypoxia activation is important for therapeutic targeting. TIP60 is recruited by HIF1α to its target genes for chromatin modification and RNA polymerase II activation (Perez-Perri et al., 2016). Both Pontin and Reptin were required for proper function of the TIP60 complex and consequently HIF1α transcription activity in this context (Figure 5D).

However, opposing roles of Pontin and Reptin have also been found for HIF1α activity (Table 2), perhaps independent of TIP60 (Lee et al., 2010, 2011): Pontin acted as an activator and Reptin as a repressor (Figure 5D). Whereas, Pontin methylation by hypoxia-induced G9a and GLP recruited p300 (a co-activator with HAT activity), Reptin methylation by G9a seems to recruit the histone deacetylase HDAC1 (Lee et al., 2010, 2011). Of interest, Pontin and Reptin were each found to regulate only a subset of hypoxia target genes that largely did not overlap with one another (Lee et al., 2010, 2011; Matias et al., 2015). This suggested that HIF1α may interact with defined partner transcription factors that required different co-activators/repressors for its transcriptional regulation, providing flexibility under different cellular/environmental contexts. Taken together, understanding these interactions could provide better and more specific targeting strategies for cancer therapy.

#### Role in p53 regulation

p53 is a transcription factor that has been studied extensively for its tumor suppression capabilities (Brown et al., 2009). Mutations in p53 that lead to the development of tumorigenesis are a common feature in cancer (Muller and Vousden, 2013). These can result from a single substitution in its amino acid sequence, which enables p53 to attain new properties that promote proliferation, metastasis and cell transformation in addition to the loss of its tumor suppressing functions (Muller and Vousden, 2013; Zhao et al., 2015). Thus, such tumor promoting p53 is termed gain-of-function mutant p53 (mutp53 GOF) (Muller and Vousden, 2013). Pontin was recently found to interact with mutp53 GOF and regulate its transcriptional activity for a subset of genes (Figure 5E; Table 2) (Zhao et al., 2015). This interaction promoted mutp53 GOF-mediated cell migration, invasion, and clonogenic potential in an ATPase dependent manner (Zhao et al., 2015).

Reptin was found to interact with wild-type p53 and suppress its anti-tumor activity (Table 2) through an interaction with anterior gradient-2 (AGR2) protein, a potent inhibitor of p53-mediated transcription that promotes cancer cell proliferation, survival, and metastasis (Figure 5E) (Maslon et al., 2010; Gray et al., 2013; Ocak et al., 2014; Clarke et al., 2016). Reptin can also inhibit p53 through repressing transcription of p14^ARF^ (alternate reading frame of CDKN2A) (Figure 5E) (Xie et al., 2012). p14^ARF^ is a tumor suppressor that acts in both p53-dependent and -independent manner (Ozenne et al., 2010). In the context of p53, p14^ARF^ binds to and inactivates MDM2, which in turn promote the stabilization and activation of p53 (Sherr and Weber, 2000; Xie et al., 2012). Thus, as an inhibitor of p14^ARF^, Reptin promotes the proliferation of cancer cells.

#### Role in NF-κB regulation

Nuclear factor-κB (NF-κB) is a family of dimeric transcription factors (p50, p52, RelA/p65, c-Rel, and RelB) activated by cellular stimuli such as oxidative stress, viral/bacterial antigen, and cytokines including TNFα and IL-1β (Moynagh, 2005). Their target genes control processes such as inflammation, cell proliferation, and cell survival (Tergaonkar, 2006). Thus, if constitutively active, unhealthy/genomically unstable cells that should normally die of apoptosis would remain in the population and lead to tumor development.

In the canonical pathway, NF-κB heterodimers are bound by IκB proteins, which sequester them in the cytoplasm and keep these transcription factors inactivated (Figure 5F) (Gilmore, 2006). A stimulus will activate the IKK (IκB kinase) complex which consists of IKKα, IKKβ, and NEMO (NF-κB essential modulator, also known as IKKγ) (Scheidereit, 2006). IKK is activated by monoubiquitination then phosphorylates the IκB inhibitor and causes its eventual degradation. This allows NF-κB to translocate into the nucleus for its function (Scheidereit, 2006).

Within the cytoplasm, RPAP3 of the R2TP complex was found to bind and regulate NEMO of the IKK complex (Shimada et al., 2011). RPAP3 binding inhibited the monoubiquitination of NEMO, which prevented the activation of the IKK complex (Figure 5F) (Shimada et al., 2011). This leads to the repression of NF-κB transcription. Whether Pontin or Reptin functions together with RPAP3 as the R2TP complex in this context is uncertain.

Pontin and Reptin were seen to regulate NF-κB p65 transcription antagonistically (Table 2), where Pontin rescued Reptin repression of transcription of p65 in reporter assays (Qiu et al., 2015). The authors suggested that Reptin repression was mediated in part through interaction with p65 in the cytoplasm and perhaps prevented degradation of the regulatory element, IκB-α (Figure 5F) (Qiu et al., 2015). IκB-α binds to and masks the nuclear localization signal of NF-κB, sequestering p65 in the cytoplasm, and thus downregulating its transcriptional activity (Tergaonkar, 2006).

Pontin bound to TIP60 was thought to co-activate a subset of NF-κB targets in response to IL-1β, including metastasis suppressor KAI1 (Kim et al., 2005, 2006; Rowe et al., 2008). In normal cells, IL-1β induces the displacement of the NCoR/TAB2 co-repressor complex (consisting of NCoR, TAB2, MEKK1, and HDAC3) that normally binds p50 (Figure 5F) (Rowe et al., 2008). This allows the recruitment and binding of co-activators Bcl3 and the Pontin-TIP60 complex, consequent acetylation at histones H3 and H4, thus leading to transcriptional activation (Kim et al., 2005).

However, Reptin in complex with β-catenin was found as a co-repressor of the same set of genes. β-catenin is a gene transcription regulator involved in the Wnt signaling pathway (described in the following section) (Kim et al., 2005) (Figure 5F). In metastatic cells, increased β-catenin expression decreases TIP60 expression and prevents binding of the co-activator complex. β-catenin with Reptin form a co-repressor complex that binds p50 (Kim et al., 2005). Repression of KAI1 expression by the Reptin-β-catenin complex was thought to occur in part through recruitment of histone deacetylase HDAC1, which required Reptin sumoylation at K456 (Figure 5F) (Kim et al., 2006). Desumoylation of Reptin by SENP-1 prevented the association with HDAC1 and decreased nuclear localization of Reptin, allowing Pontin/TIP60 to bind and activate transcription (Kim et al., 2006).

In general, the two ATPases through their respective complexes were shown to bind NF-κB transcription factor p50 at the promoter region of KAI1 in a mutually exclusive manner (Kim et al., 2005, 2006). Thus, this represents another instance where Pontin and Reptin seem to act independently of each other, and even antagonistically.

#### Role in β-catenin regulation

β-catenin is another transcriptional regulator highly involved in oncogenesis. In the canonical Wnt-signaling pathway, it interacts with the LEF/TCF (lymphoid enhancing factor/T-cell factor) family of transcription factors to activate numerous genes involved in proliferation and survival (Macdonald et al., 2009). The pathway is often found activated in a variety of cancers, either through activating mutations in β-catenin itself or in proteins involved in Wnt-signaling (Morin, 1999; Macdonald et al., 2009). Pontin and Reptin possess opposing regulatory functions in this pathway (Bauer et al., 1998, 2000; Yakulov et al., 2013) (Table 2). The ATPases were shown *in vitro* to directly interact with β-catenin in the same region, and thus might exhibit competitive binding (Bauer et al., 1998, 2000). Similar to previous cases, such as for H1Fα- and NF-κB-dependent transcription, repression by Reptin is mediated by recruitment of HDAC. Here specifically, Reptin sumoylation was shown to be important for recruiting HDAC and consequently repressing the transcriptional activity of canonical β-catenin targets such as cyclin D1 (Figure 5G) (Bauer et al., 2000). Whether Pontin recruits TIP60 or other HATs for its co-activating activities on β-catenin remains to be investigated. Recently, an anti-apoptotic protein c-FLIP_L_ (cellular FLICE-like inhibitory protein) was found to promote activation of β-catenin-dependent transcription by Pontin (Zhang et al., 2017). c-FLIP_L_ increased binding of Pontin at target gene promoters by binding to Pontin using its DED (death-effector domain) (Zhang et al., 2017).

The role of Pontin and Reptin in β-catenin-LEF/TCF mediated transcription may also be inhibited by other protein(s). For instance, Hint1 (histidine triad nucleotide-binding protein 1) was found to suppress Pontin activation of β-catenin transcription, and APPL1/2 (adaptor proteins containing pleckstrin homology domain, phosphotyrosine binding domain, and leucine zipper domain) were shown to relieve the repression of transcription by Reptin (Figure 5G) (Weiske and Huber, 2005; Rashid et al., 2009). Hint1 is implicated in transcription regulation and growth control, and the HIT family of proteins, to which Hint1 belongs, is often found inactive in many carcinomas (Weiske and Huber, 2006). APPL1/2 are effectors of the small GTPase Rab5 and function in early steps of endocytosis (Rashid et al., 2009). Whereas, Hint1 prevented Pontin to Pontin interactions, APPLs reduced the association between Reptin, HDAC and β-catenin (Weiske and Huber, 2005; Rashid et al., 2009).

#### Role in the regulation of other transcription factors

Oct4, one of the main ESC (embryonic stem cell)-specific transcription factors, is essential for regulating embryonic development and the self-renewing property of ESCs (Shi and Jin, 2010). Pontin acts as a transcriptional co-activator of Oct4 for both the expression of genes required for ESC maintenance and for lincRNAs (long non-coding RNAs) that repress the lineage differentiation program in ESCs through methyltransferases such as Ezh2 (Figure 5H) (Boo et al., 2015) (Table 2). Pontin activation of Oct4 targets is thought to be mediated by recruitment of p300 acetyltransferase (Boo et al., 2015). Reptin was also found to maintain pluripotency of ESCs, perhaps acting in complex with Pontin (Do et al., 2014) (Table 2).

Using proteomics, EVI1 (Ecotropic viral integration site-1), C/EBP (CCAAT/enhancer-binding protein) alpha and beta, which are transcription factors, were found to interact with Pontin and Reptin (Figure 5H) (Bard-Chapeau et al., 2013; Cirilli et al., 2016). EVI1 is an oncogenic transcription factor that is often overexpressed in cancers such as myeloid leukemia and epithelial cancers, while C/EBPα and β regulate processes such as cell proliferation, apoptosis and transformation (Bard-Chapeau et al., 2013; Cirilli et al., 2016). Though identified, the functional role and molecular mechanism of Pontin/Reptin interactions with these transcription factors are not known.

### Role of pontin/reptin in the DNA damage and repair

Genomic instability is a hallmark of cancer. DNA damage response (DDR) and repair pathways are typically induced under these conditions (Jeggo et al., 2016). Failure to properly repair DNA damage allows accumulation of damage and results in genomic instability, promoting development of cancer (Ciccia and Elledge, 2010; O'Connor, 2015). On the other hand, the cytotoxicity of the DNA damage has been largely exploited for chemotherapy, but not without significant collateral damage and side effects (Deans and West, 2011; O'Connor, 2015). Thus recently, DDR has been explored for more targeted chemotherapy.

It is well-known that Pontin and Reptin are involved in DNA damage response due to their participation in protein complexes that are major players in this process (Grigoletto et al., 2011; Matias et al., 2015). Such complexes include the TIP60 complex mentioned previously and the chromatin remodeling complex INO80. Pontin and Reptin have recently been shown to interact with transcription factors RUNX2 (Runt-related transcription factor 2) and YY1 (Ying-Yang 1) for processes involved in DNA damage response (Wu et al., 2007; Lopez-Perrote et al., 2014; Yang et al., 2015). More recently, Pontin and Reptin were also found to be important for the stability of the Fanconi anemia (FA) core complex that functions in interstrand-crosslink (ICL) repair (Rajendra et al., 2014). The two ATPases participate in these processes together as a heterohexamer and/or independently to provide a broad spectrum of responses to the various circumstances and stresses that a cell encounters.

#### Role in TIP60 complex—H2AX regulation

Phosphorylation of histone variant H2AX on Ser139 is one of the earliest events following DNA damage (O'Connor, 2015). Its abundant signal allows it to act as a sensitive marker for DNA damage and the repair that follows (Ciccia and Elledge, 2010). As subunits of the TIP60 complex, Pontin and Reptin are highly involved in the regulation of this signal.

**Figure 6:**
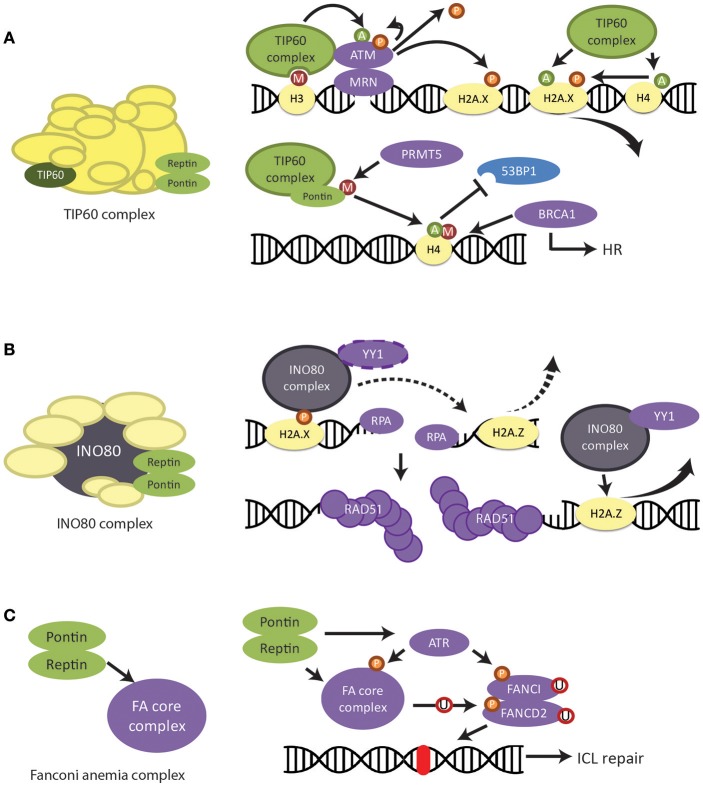
Regulation of DNA damage response and repair by Pontin/Reptin. **(A)** TIP60 complex. Pontin and Reptin participate in the TIP60 complex to promote DNA damage signaling and repair. UPPER—TIP60 regulates the activation and removal of phospho-H2AX signal through acetylation of various proteins. LOWER—Pontin methylation by PRMT5 is required for the acetylation of H4 by TIP60. This acetylation prevents the binding of 53BP1 to the methylation site on H4 but allows BRCA1 to bind, thus promoting homologous recombination. **(B)** INO80 complex. Pontin and Reptin participate in the INO80 complex to promote homologous recombination. The INO80 complex is recruited to the DNA damage site by phospho-H2AX. It is involved in RPA-mediated DNA end resection as well as RAD51-mediated DNA strand invasion, likely by the removal of H2A.Z and in complex with YY1. **(C)** Fanconi Anemia core complex. Pontin and Reptin stabilize the FA core complex either directly or through its stabilizing activity of the ATR kinase, which then activates the FA pathway.

After DNA damage, histone H3 methylation site is exposed and the MRN complex (consisting of Mre11, Rad50, and Nbs1 proteins) binds to the damaged site. MRN then recruits TIP60 in complex with checkpoint kinase ATM and facilitates the interaction between TIP60 and methylated H3 (Figure 6A) (Sun et al., 2009). This interaction upregulates the acetyltransferase activity of TIP60. ATM is activated through acetylation by TIP60 and autophosphorylates for further activation (Sun et al., 2005). ATM is then able to phosphorylate histone H2AX and a host of DNA damage proteins, regulating downstream signaling (Ciccia and Elledge, 2010) (Figure 6A).

Depletion of Pontin after DNA damage increased the amount and lifetime of phosphorylated H2AX, which could be mimicked by TIP60 depletion (Jha et al., 2008). Since Pontin is required for the histone acetyltransferase activity of TIP60, this suggested that Pontin in complex with TIP60 was also important for the removal of phospho-H2AX (Kusch et al., 2004; Jha et al., 2013) (Figure 6A). In agreement, Ikura et al. (2007) found that TIP60 acetylation of H2AX mediates its release from chromatin (Figure 6A). On the other hand, TIP60 acetylation of H4 is also required for curbing the phospho-H2AX signal (Jha et al., 2008).

Conflicting results have been reported regarding Reptin depletion. Whereas, Ni et al. (2009) found that Reptin depletion increased H2AX phosphorylation following UV irradiation in HeLa cells, Raymond et al. (2015) showed that etoposide or γ irradiation of HuH7 and Hep3B cells, which produced double strand breaks (DSBs), led to reduced phosphorylation of H2AX upon Reptin depletion, and thus resulted in defective repair. Further studies are needed to explain whether these differences are due to the nature of the DNA lesion, source of damage or cell type specificity. Raymond et al. (2015) also found that DSB repair was regulated by Reptin in part through stabilizing DNA-PKcs (a member of the PIKK family). Thus, overexpression of Reptin in chemoresistant ovarian and breast cancers could confer higher DNA damage repair abilities and partly explain their resistance to therapy (Yang et al., 2012).

#### Role in TIP60 complex—homologous recombination

DSB is the most toxic and dangerous type of DNA damage, as it can lead to loss of genetic material if left unresolved (Ciccia and Elledge, 2010). The two main repair strategies for DSBs are homologous recombination (HR) and non-homologous end joining (NHEJ) (Clarke et al., 2017). As their names suggests, HR uses a template DNA (the sister chromatid) for repair, and thus is less error prone, while NHEJ is an inaccurate process known to lead to genomic instability and thus cancer susceptibility (Ciccia and Elledge, 2010; Jeggo et al., 2016). However, HR is restricted to late S/G2 phase due to its requirement for a template sequence, whereas NHEJ can occur any time during the cell cycle (Clarke et al., 2017). The main factor in determining which repair pathway is used depends on the extent of DNA end processing, which is controlled by the 53BP1 (p53 binding protein 1)-containing complex that protects the ends from over-processing (Tang et al., 2013). The HR pathway requires the dissociation of 53BP1 for extensive end resection by specialized machinery, which is facilitated by the recruitment of BRCA1 (breast cancer early onset 1) (Kusch et al., 2004; Tang et al., 2013). Competitive binding of BRCA1 and 53BP1 at DSB sites on the chromatin determines the pathway choice between HR and NHEJ: BRCA1 binding promotes HR and 53BP1 binding promotes NHEJ (Clarke et al., 2017).

Pontin and Reptin were found to be involved in HR through both TIP60 and INO80 complexes. TIP60 acetylates histone H4 at K16, disrupting the interaction between the H4K16 residue and 53BP1 (Sun et al., 2009; Tang et al., 2013). This, in combination with recruitment of TIP60 complex subunit MBTD1 to the methylation site on H4 at K20 displaces 53BP1 from the histone tail (Figure 6A) (Jacquet et al., 2016). It was recently found that PRMT5 (protein arginine methyltransferase 5) methylated Pontin at R205 and that this was required for the acetyltransferase activity of TIP60 and, consequently, for the mobilization of 53BP1 (Figure 6A) (Clarke et al., 2017). Thus, it was not surprising that TIP60-deficiency led to impaired HR and conferred sensitivity to DNA-damaging anticancer therapy based on poly ADP ribose polymerase (PARP) inhibition, a phenotype mimicked by Pontin depletion (Tang et al., 2013).

#### Role in INO80 complex

It was known that INO80 also facilitates HR, but the molecular mechanism had been unclear (Wu et al., 2007; Tsukuda et al., 2009; Gospodinov et al., 2011). Gospodinov et al. (2011) found that INO80 mediates resection of double-strand break ends and is required for the formation of replication protein A (RPA) foci. RPA functions to prevents single stranded DNA created during resection from forming secondary structures or winding back onto itself (Ciccia and Elledge, 2010). Alatwi and Downs (2015) reported that depletion of INO80 after DNA damage led to defective RAD51 foci formation, a phenotype also seen with TIP60 depletion. RAD51 is recruited to resected ends of the damaged DNA and is the primary mediator of strand invasion and recombination for the HR pathway (Ciccia and Elledge, 2010). The role of INO80 in both processes might be to remove histone variant H2A.Z for the resolution of repair in complex with YY1 (Figure 6B) (Alatwi and Downs, 2015). As subunits of the INO80 complex, Pontin and Reptin were also seen to accumulate at DSBs (Alatwi and Downs, 2015). Their ATPase activity was required for the formation of RAD51 foci, through direct interaction and in cooperation with the YY1 transcription factor (Wu et al., 2007; Lopez-Perrote et al., 2014).

#### Role in Fanconi anemia DNA repair pathway

Pontin and Reptin were shown to be involved in yet another DNA repair pathway, the Fanconi anemia (FA) pathway, responsible for the repair of interstrand crosslinks (Deans and West, 2011). It was recently demonstrated that Pontin and Reptin interacted directly with the FA core complex, and regulated the abundance of the FA subunits on both the protein and mRNA levels (Rajendra et al., 2014). Depletion of these two ATPases resulted in sensitivity to DNA crosslinking agents, chromosome aberrations and defective FA pathway activation (Rajendra et al., 2014).

Pontin and Reptin can regulate the FA core complex either directly or through maintaining the stability of its upstream activator the serine/threonine-protein kinase ATR (Figure 6C) (Rajendra et al., 2014), which is a member of the PIKK family. ATR also activates the FANCI and FANCD2 dimer through phosphorylation (Deans and West, 2011). This activation is completed by the monoubiquitination of the dimer by the FA core complex (Ceccaldi et al., 2016). The dimer participates in subsequent recruitment of nucleases and other proteins important for interstrand crosslink repair (Rajendra et al., 2014; Ceccaldi et al., 2016).

### Role of pontin/reptin in epithelial-mesenchymal transition (EMT)

The role of Pontin and Reptin in cell migration and invasion has recently been investigated although is not yet well-elucidated (Ren et al., 2013; Taniuchi et al., 2014; Zhang et al., 2015; Breig et al., 2016). As mentioned above, Pontin and Reptin were originally thought of as nuclear proteins, however, accumulating research demonstrated their cytoplasmic localization as well, ranging from partial to predominantly cytoplasmic (Grigoletto et al., 2011). This was also recently found to have a clinical significance, perhaps acting through the epithelial to mesenchymal transition (EMT) pathway.

#### Cytoplasmic localization of pontin/reptin

Localization of Pontin and Reptin in the cytoplasm seems to be a common marker for cancer metastasis and involvement in cell migration. High cytoplasmic expression of the proteins was correlated with poor prognosis and metastatic progression in patients with HCC and RCC (Ren et al., 2013; Zhang et al., 2015; Breig et al., 2016). Cytoplasmic localization of Pontin was also reported in human colorectal cancer (CRC) and lymphoma tissue sections, as well as in PDAC cells, embryonic stem cells (ESCs), and HeLa cells; while Reptin cytoplasmic localization was found in HEK293 cells, HeLa cells and adipocytes (Makino et al., 1998; Mizuno et al., 2006; Lauscher et al., 2007; Ni et al., 2009; Xie et al., 2009; Taniuchi et al., 2014; Baron et al., 2016). This suggested the presence of functions of Pontin and Reptin specific to the cytoplasm, outside of their roles in chromatin remodeling, DNA damage response, and transcriptional regulation within the nucleus.

Depletion of Pontin and Reptin in RCC cells (A498, 786-O), where their expression was predominantly cytoplasmic, significantly inhibited cell migration and invasion ability (Ren et al., 2013; Zhang et al., 2015). A similar phenotype was observed upon Pontin/Reptin silencing in many other cancer models such as prostate cancer (LNCap), HCC (HuH7, Hep3B), PDAC (S2-013), and hypoxia treated breast cancer cells (MCF7) (Kim et al., 2006; Rousseau et al., 2007; Lee et al., 2010; Taniuchi et al., 2014). However, the molecular mechanism by which this occurs is currently unclear.

#### Role in regulating EMT-associated cellular events

**Figure 7:**
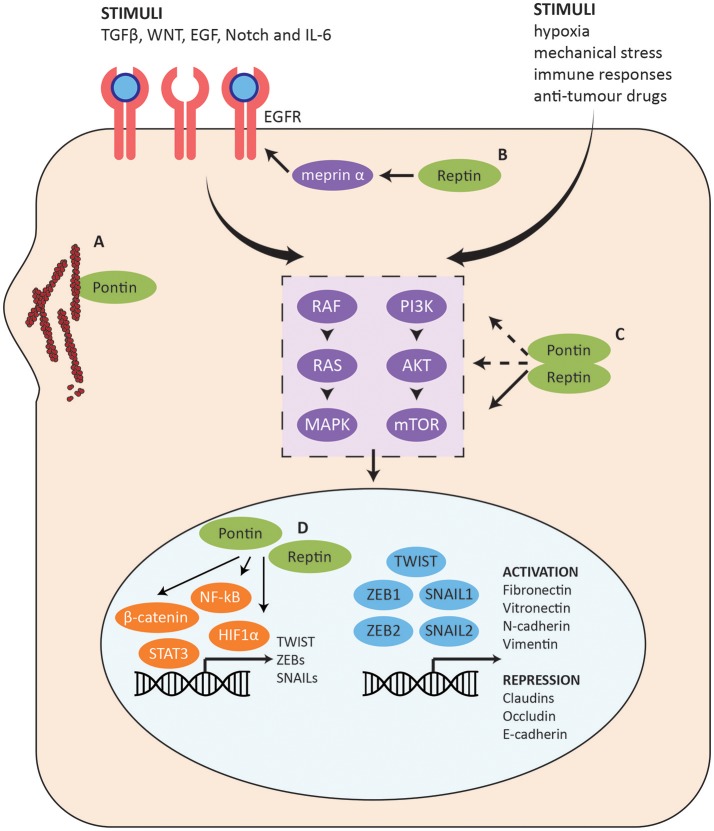
Role of Pontin/Reptin in the EMT pathway. Summary of the EMT pathway and the stages at which Pontin and Reptin potentially function to promote cell invasion and migration. **(A)** Pontin promotes F-actin polymerization and G-actin local concentration. **(B)** Reptin promotes EGFR signaling through regulating the mRNA and protein expression of meprin α. **(C)** Pontin and Reptin activate PI3K-AKT-mTOR intracellular signaling at multiple stages. **(D)** Pontin and Reptin interact with cell survival/proliferation transcription factors (shown in orange) to promote transcription of many genes involved in metastasis, which may include several master EMT transcription factors (shown in blue).

Analysis of human RCC tissue samples showed an overexpression of Pontin and decreased expression of E-cadherin (an epithelial marker) compared to normal renal tissue (Zhang et al., 2015). Loss of E-cadherin expression is a hallmark of EMT, followed by disassembly of epithelial cell-cell junctions, loss of apical-basal polarity, reorganization of cortical cytoskeleton and increased cell mobility (Figure 7) (Lamouille et al., 2014). This allows tumor cells that have undergone EMT to disseminate to distant sites, become resistant to apoptosis and senescence, and act as cancer stem cells (CSCs) (Marcucci et al., 2016). Mounting evidence showed that EMT is a crucial mechanism for malignant transformation and metastatic progression (Marcucci et al., 2016). Intriguingly, Pontin depletion dramatically increased E-cadherin expression, which suggested that Pontin/Reptin may also promote cell migration and invasion through the EMT pathway (Zhang et al., 2015). Furthermore, decreased vimentin expression (a mesenchymal marker) was observed after silencing either Pontin or Reptin, further supporting this hypothesis (Zhang et al., 2015).

In addition to the changes in expression profiles of various cell adhesion and cell junction genes, EMT is also helped by changes in the cell matrix and cytoskeleton through reorganization of actin and intermediate filaments. PDAC is the most common type of pancreatic cancer, and one of the most difficult to treat due to its aggressive and highly metastatic nature. Taniuchi et al. (2014) found that Pontin promotes invasiveness and migration of PDAC cells through a direct interaction with actin filaments at cell protrusions (Figure 7A). Pontin mediated actin polymerization by binding filamentous-actin (F-actin), which enhanced elongation of existing actin filaments. Globular-actin (G-actin) is the monomeric building block for F-actin and sufficient concentration is needed for efficient assembly of filaments. Even though Pontin did not interact with G-actin, it increased the localization of G-actin at cell protrusions, allowing increased F-actin structures and actin-based motility. Knockdown of Pontin decreased peripheral actin rearrangements, thus inhibiting formation of cell protrusions (Taniuchi et al., 2014). This in turn repressed the motility and invasiveness of PDAC cells, which can prove to be valuable for therapeutic targeting.

#### Role in EMT pathway signaling

The cellular changes in EMT are controlled by a complex underlying molecular mechanism and crosstalk between many signaling pathways such as TGFβ, WNT, EGF, Notch, and IL-6 (Figure 7) (Lamouille et al., 2014). Reptin may enhance activation of these receptors through its interaction with meprin α (MEP1A), a secreted metalloproteinase with pro-angiogenic and pro-migratory activity (Lottaz et al., 2011; Minder et al., 2012). Many of its targets are highly relevant for cancer and the EMT pathway (Broder and Becker-Pauly, 2013; Breig et al., 2016). Meprin α mediated the transactivation of the EGFR signaling in colorectal cancer and has a possible role in invasion and metastatic dissemination (Minder et al., 2012). In the HCC context, Breig et al. (2016) found meprin α to be a downstream mediator for Reptin-dependent migration and cell invasion (Figure 7B). Exogenous meprin α was able to restore migration and invasion capabilities but not proliferation in Reptin-silenced cells. Reptin also regulated both mRNA and protein expression of meprin α, though the mechanism has yet been elucidated. Furthermore, the expression of Reptin and meprin α were correlated in patient samples. Expression of either proteins were also independently found to be correlated with poor differentiation and low post-operative survival, supporting their potential involvement in EMT (Breig et al., 2016).

In a cancer context, stimuli such as hypoxia and mechanical stress from the tumor microenvironment and/or overexpression of pathway components can work cooperatively to induce EMT (Marcucci et al., 2016). These will activate a number of intracellular signaling pathways including the PI3K-AKT-mTOR pathway and the RAS-RAF-MAPK pathway that are also highly involved in sustaining cancer cell growth and proliferation (Figure 7C) (Lamouille et al., 2014; Marcucci et al., 2016). Pontin and Reptin can potentially promote EMT through the PI3K-AKT-mTOR pathway since they have been found to be important for mTORC1 stabilization and activation (Kim et al., 2013). Pontin silencing also reduced the levels of AKT protein in lung adenocarcinoma, which suggests that it may also function in the upstream activation of the pathway or can stabilize AKT as well (Figure 7C) (Yuan et al., 2016).

#### Role in EMT transcriptional regulation

Transcription factors such as NF-κB, STAT3, H1F1α, and β-catenin are upregulated downstream of the intracellular signaling pathways activated in EMT, which then induces the expression and activation of a pool of EMT-promoting master transcription factors such as TWIST, SNAILs, and ZEBs (Figure 7D) (Lamouille et al., 2014). These directly control the expression of genes associated with epithelial and mesenchymal phenotypes including E-cadherin, fibronectin, and vimentin (Lamouille et al., 2014).

Pontin and Reptin have been found to regulate β-catenin transcription with opposing effects: Pontin enhancing transcription and Reptin repressing it (Figure 7D) (Bauer et al., 2000). Nuclear β-catenin expression significantly decreased following Pontin depletion in RCC, supporting a hypothesis that during oncogenesis, Pontin may upregulate β-catenin transcription targets by relocating β-catenin from the adherens junctions to the nucleus, thus increasing transcription of oncogenes such as MYC (Ren et al., 2013). This is consistent with Lauscher et al.'s (2007) observation where nuclear co-localization of Pontin and β-catenin was correlated with progression of colorectal cancer. In addition, Kim et al. (2005) showed the importance of Reptin in repressing the anti-metastatic gene KAI1. Pontin and Reptin were also shown to regulate transcription in the hypoxia pathway, as well as in promoting expression of interferon-stimulated genes (Figure 7D) (Lee et al., 2010, 2011; Gnatovskiy et al., 2013; Perez-Perri et al., 2016).

## Drug targeting

As describe above, Pontin and Reptin are clearly important proteins for the proliferation and survival of cells. Through their diverse cellular functions, they are highly relevant for the progression of cancer, and this makes them novel therapeutic anticancer drug targets. Conditional silencing of Reptin in xenografts of HCC in mice led to arrest of tumor development and even regression of tumors in several mice likely through tumor cell senescence (Menard et al., 2010). Mice with conditional hemizygous knockout of Pontin resulted in significantly smaller tumor formation 6 months after induction of cancer (Bereshchenko et al., 2012). However, the same mice when examined at 9–12 months showed accelerated progression of cancer and tumor formation, suggesting that long-term Pontin inhibition may pose unforeseen risks *in vivo* (Bereshchenko et al., 2012). This is to be expected due to its many fundamental roles for normal cells.

Preliminary *in silico* drug screening for Pontin ATPase inhibitors was able to identify several novel compounds using both molecular docking and *in vitro* ATPase assays (Elkaim et al., 2012). Three of these compounds showed anti-proliferative activities on tumor cells. Using the same method on a pool of pre-existing molecules, two specific compounds were identified that competitively bound to the ATP-binding pocket (Elkaim et al., 2014). One compound induced apoptosis as well as necrosis in cellular assays and thus has the potential to be developed further as a therapeutic. In addition, high-throughput screening by Daiichi-Sankyo Co. LTD. in Japan also identified compounds that were selective Pontin/Reptin ATPase inhibitors (Patent WO2015125786A1). These compounds inhibited tumor cell proliferation *in vitro* as well as demonstrated anti-tumor activities *in vivo* with human xenografts in mice.

There is accumulating evidence that Pontin and Reptin functions are tightly regulated on multiple levels, including transcription, oligomeric state, subcellular localization, and interacting partners. Recent studies suggested that different post-translation modifications such as methylation, sumoylation, and phosphorylation may partially modulate the specificity of their roles. Thus, developing drugs to target these post-translational modifications and or their specific interactions may be more beneficial and less toxic than inhibiting the overall activities of these proteins.

## Concluding remarks

Consistent with the overexpression of Pontin and Reptin in many cancer types, the two AAA+ proteins are found to regulate many fundamental cellular pathways involved in cell proliferation and survival. These include the assembly of macromolecular complexes as part of the R2TP complex, regulation of cell cycle checkpoint and mitosis, regulation of oncogenic transcription factors, regulation of DNA damage response as well as repair, and promotion of epithelial to mesenchymal transition among others. Much is still unknown about the molecular mechanism that Pontin and Reptin use to facilitate these processes or the repertoire of interactors involved. It is, therefore, crucial to shed further light into the roles that Pontin and Reptin perform in the cell to accelerate the discovery of novel therapeutics for various types of cancer.

## Author contributions

Y-QM and WAH wrote and edited the manuscript.

### Conflict of interest statement

The authors declare that the research was conducted in the absence of any commercial or financial relationships that could be construed as a potential conflict of interest.

## References

[B1] Alatwi H. E. Downs J. A. (2015). Removal of H2A.Z by INO80 promotes homologous recombination. EMBO Rep. 16, 986–994. 10.15252/embr.201540330 26142279 PMC4552491

[B2] Bakkenist C. J. Kastan M. B. (2004). Initiating cellular stress responses. Cell 118, 9–17. 10.1016/j.cell.2004.06.023 15242640

[B3] Bard-Chapeau E. A. Gunaratne J. Kumar P. Chua B. Q. Muller J. Bard F. A.. (2013). EVI1 oncoprotein interacts with a large and complex network of proteins and integrates signals through protein phosphorylation. Proc. Natl. Acad. Sci. U.S.A. 110, E2885–E2894. 10.1073/pnas.1309310110 23858473 PMC3732971

[B4] Baretic D. Williams R. L. (2014). PIKKs–the solenoid nest where partners and kinases meet. Curr. Opin. Struct. Biol. 29, 134–142. 10.1016/j.sbi.2014.11.003 25460276

[B5] Baron B. W. Baron R. M. Baron J. M. (2016). The relationship between RUVBL1 (Pontin, TIP49, NMP238) and BCL6 in benign and malignant human lymphoid tissues. Biochem. Biophys. Rep. 6, 1–8. 10.1016/j.bbrep.2016.02.006 27066592 PMC4822715

[B6] Battle D. J. Kasim M. Yong J. Lotti F. Lau C. K. Mouaikel J.. (2006). The SMN complex: an assembly machine for RNPs. Cold Spring Harb. Symp. Quant. Biol. 71, 313–320. 10.1101/sqb.2006.71.001 17381311

[B7] Bauer A. Chauvet S. Huber O. Usseglio F. Rothbacher U. Aragnol D.. (2000). Pontin52 and reptin52 function as antagonistic regulators of beta-catenin signalling activity. EMBO J. 19, 6121–6130. 10.1093/emboj/19.22.6121 11080158 PMC305835

[B8] Bauer A. Huber O. Kemler R. (1998). Pontin52, an interaction partner of beta-catenin, binds to the TATA box binding protein. Proc. Natl. Acad. Sci. U.S.A. 95, 14787–14792. 10.1073/pnas.95.25.14787 9843967 PMC24527

[B9] Bellosta P. Hulf T. Balla Diop S. Usseglio F. Pradel J. Aragnol D.. (2005). Myc interacts genetically with Tip48/Reptin and Tip49/Pontin to control growth and proliferation during Drosophila development. Proc. Natl. Acad. Sci. U.S.A. 102, 11799–11804. 10.1073/pnas.0408945102 16087886 PMC1187951

[B10] Berasain C. (2010). New therapeutic targets in HCC: reptin ATPase and HCC senescence. J. Hepatol. 52, 633–634. 10.1016/j.jhep.2010.01.020 20334944

[B11] Bereshchenko O. Mancini E. Luciani L. Gambardella A. Riccardi C. Nerlov C. (2012). Pontin is essential for murine hematopoietic stem cell survival. Haematologica 97, 1291–1294. 10.3324/haematol.2011.060251 22371176 PMC3436228

[B12] Bizarro J. Charron C. Boulon S. Westman B. Pradet-Balade B. Vandermoere F.. (2014). Proteomic and 3D structure analyses highlight the C/D box snoRNP assembly mechanism and its control. J. Cell Biol. 207, 463–480. 10.1083/jcb.201404160 25404746 PMC4242836

[B13] Bizarro J. Dodre M. Huttin A. Charpentier B. Schlotter F. Branlant C.. (2015). NUFIP and the HSP90/R2TP chaperone bind the SMN complex and facilitate assembly of U4-specific proteins. Nucleic Acids Res. 43, 8973–8989. 10.1093/nar/gkv809 26275778 PMC4605303

[B14] Bjorklund M. Taipale M. Varjosalo M. Saharinen J. Lahdenpera J. Taipale J. (2006). Identification of pathways regulating cell size and cell-cycle progression by RNAi. Nature 439, 1009–1013. 10.1038/nature04469 16496002

[B15] Boo K. Bhin J. Jeon Y. Kim J. Shin H. J. Park J. E.. (2015). Pontin functions as an essential coactivator for Oct4-dependent lincRNA expression in mouse embryonic stem cells. Nat. Commun. 6:6810. 10.1038/ncomms7810 25857206 PMC4403444

[B16] Boulon S. Bertrand E. Pradet-Balade B. (2012). HSP90 and the R2TP co-chaperone complex: building multi-protein machineries essential for cell growth and gene expression. RNA Biol. 9, 148–154. 10.4161/rna.18494 22418846

[B17] Boulon S. Marmier-Gourrier N. Pradet-Balade B. Wurth L. Verheggen C. Jady B. E.. (2008). The Hsp90 chaperone controls the biogenesis of L7Ae RNPs through conserved machinery. J. Cell Biol. 180, 579–595. 10.1083/jcb.200708110 18268104 PMC2234240

[B18] Boulon S. Pradet-Balade B. Verheggen C. Molle D. Boireau S. Georgieva M.. (2010). HSP90 and its R2TP/Prefoldin-like cochaperone are involved in the cytoplasmic assembly of RNA polymerase II. Mol. Cell 39, 912–924. 10.1016/j.molcel.2010.08.023 20864038 PMC4333224

[B19] Breig O. Bras S. Martinez Soria N. Osman D. Heidenreich O. Haenlin M.. (2014). Pontin is a critical regulator for AML1-ETO-induced leukemia. Leukemia 28, 1271–1279. 10.1038/leu.2013.376 24342949

[B20] Breig O. Yates M. Neaud V. Couchy G. Grigoletto A. Lucchesi C.. (2016). Metalloproteinase meprin alpha regulates migration and invasion of human hepatocarcinoma cells and is a mediator of the oncoprotein Reptin. Oncotarget 8, 7839–7851. 10.18632/oncotarget.13975 27999200 PMC5352365

[B21] Broder C. Becker-Pauly C. (2013). The metalloproteases meprin alpha and meprin beta: unique enzymes in inflammation, neurodegeneration, cancer and fibrosis. Biochem. J. 450, 253–264. 10.1042/BJ20121751 23410038 PMC3573791

[B22] Brown C. J. Lain S. Verma C. S. Fersht A. R. Lane D. P. (2009). Awakening guardian angels: drugging the p53 pathway. Nat. Rev. Cancer 9, 862–873. 10.1038/nrc2763 19935675

[B23] Ceccaldi R. Sarangi P. D'andrea A. D. (2016). The Fanconi anaemia pathway: new players and new functions. Nat. Rev. Mol. Cell Biol. 17, 337–349. 10.1038/nrm.2016.48 27145721

[B24] Cheung K. L. Huen J. Houry W. A. Ortega J. (2010a). Comparison of the multiple oligomeric structures observed for the Rvb1 and Rvb2 proteins. Biochem. Cell Biol. 88, 77–88. 10.1139/O09-159 20130681 PMC2980847

[B25] Cheung K. L. Huen J. Kakihara Y. Houry W. A. Ortega J. (2010b). Alternative oligomeric states of the yeast Rvb1/Rvb2 complex induced by histidine tags. J. Mol. Biol. 404, 478–492. 10.1016/j.jmb.2010.10.003 20934430 PMC2982833

[B26] Ciccia A. Elledge S. J. (2010). The DNA damage response: making it safe to play with knives. Mol. Cell 40, 179–204. 10.1016/j.molcel.2010.09.019 20965415 PMC2988877

[B27] Cirilli M. Bereshchenko O. Ermakova O. Nerlov C. (2016). Insights into specificity, redundancy and new cellular functions of C/EBPa and C/EBPb transcription factors through interactome network analysis. Biochim. Biophys. Acta 1861, 467–476. 10.1016/j.bbagen.2016.10.002 27746211

[B28] Clarke D. J. Murray E. Faktor J. Mohtar A. Vojtesek B. Mackay C. L.. (2016). Mass spectrometry analysis of the oxidation states of the pro-oncogenic protein anterior gradient-2 reveals covalent dimerization via an intermolecular disulphide bond. Biochim. Biophys. Acta 1864, 551–561. 10.1016/j.bbapap.2016.02.011 26876500

[B29] Clarke T. L. Sanchez-Bailon M. P. Chiang K. Reynolds J. J. Herrero-Ruiz J. Bandeiras T. M.. (2017). PRMT5-dependent methylation of the TIP60 coactivator RUVBL1 is a key regulator of homologous recombination. Mol Cell 65 900–916.e907. 10.1016/j.molcel.2017.01.019 28238654 PMC5344794

[B30] Cloutier P. Poitras C. Durand M. Hekmat O. Fiola-Masson E. Bouchard A.. (2017). R2TP/Prefoldin-like component RUVBL1/RUVBL2 directly interacts with ZNHIT2 to regulate assembly of U5 small nuclear ribonucleoprotein. Nat. Commun. 8:15615. 10.1038/ncomms15615 28561026 PMC5460035

[B31] Cui F. Zan X. Li Y. Sun W. Yang Y. Ping L. (2016). Grifola frondosa glycoprotein GFG-3a arrests S phase, alters proteome, and induces apoptosis in human gastric cancer cells. Nutr. Cancer 68, 267–279. 10.1080/01635581.2016.1134599 27040446

[B32] Cvackova Z. Albring K. F. Koberna K. Ligasova A. Huber O. Raska I.. (2008). Pontin is localized in nucleolar fibrillar centers. Chromosoma 117, 487–497. 10.1007/s00412-008-0170-8 18548265 PMC2564108

[B33] Dalvai M. Bellucci L. Fleury L. Lavigne A. C. Moutahir F. Bystricky K. (2013). H2A.Z-dependent crosstalk between enhancer and promoter regulates cyclin D1 expression. Oncogene 32, 4243–4251. 10.1038/onc.2012.442 23108396

[B34] Dang C. V. (2012). MYC on the path to cancer. Cell 149, 22–35. 10.1016/j.cell.2012.03.003 22464321 PMC3345192

[B35] David-Morrison G. Xu Z. Rui Y. N. Charng W. L. Jaiswal M. Yamamoto S.. (2016). WAC regulates mTOR Activity by acting as an adaptor for the TTT and pontin/reptin complexes. Dev. Cell 36, 139–151. 10.1016/j.devcel.2015.12.019 26812014 PMC4730548

[B36] Deans A. J. West S. C. (2011). DNA interstrand crosslink repair and cancer. Nat. Rev. Cancer 11, 467–480. 10.1038/nrc3088 21701511 PMC3560328

[B37] Dehan E. Ben-Dor A. Liao W. Lipson D. Frimer H. Rienstein S.. (2007). Chromosomal aberrations and gene expression profiles in non-small cell lung cancer. Lung Cancer 56, 175–184. 10.1016/j.lungcan.2006.12.010 17258348

[B38] Desjarlais R. Tummino P. J. (2016). Role of histone-modifying enzymes and their complexes in regulation of chromatin biology. Biochemistry 55, 1584–1599. 10.1021/acs.biochem.5b01210 26745824

[B39] Do E. K. Cheon H. C. Jang I. H. Choi E. J. Heo S. C. Kang K. T.. (2014). Reptin regulates pluripotency of embryonic stem cells and somatic cell reprogramming through Oct4-dependent mechanism. Stem Cells 32, 3126–3136. 10.1002/stem.1827 25185564

[B40] Dobreva I. Fielding A. Foster L. J. Dedhar S. (2008). Mapping the integrin-linked kinase interactome using SILAC. J. Proteome Res. 7, 1740–1749. 10.1021/pr700852r 18327965

[B41] Domoto T. Pyko I. V. Furuta T. Miyashita K. Uehara M. Shimasaki T.. (2016). Glycogen synthase kinase-3beta is a pivotal mediator of cancer invasion and resistance to therapy. Cancer Sci. 107, 1363–1372. 10.1111/cas.13028 27486911 PMC5084660

[B42] Ducat D. Kawaguchi S. Liu H. Yates J. R. III. Zheng Y. (2008). Regulation of microtubule assembly and organization in mitosis by the AAA+ ATPase Pontin. Mol. Biol. Cell 19, 3097–3110. 10.1091/mbc.E07-11-1202 18463163 PMC2441676

[B43] Dugan K. A. Wood M. A. Cole M. D. (2002). TIP49, but not TRRAP, modulates c-Myc and E2F1 dependent apoptosis. Oncogene 21, 5835–5843. 10.1038/sj.onc.1205763 12185582

[B44] Elkaim J. Castroviejo M. Bennani D. Taouji S. Allain N. Laguerre M.. (2012). First identification of small-molecule inhibitors of Pontin by combining virtual screening and enzymatic assay. Biochem. J. 443, 549–559. 10.1042/BJ20111779 22273052

[B45] Elkaim J. Lamblin M. Laguerre M. Rosenbaum J. Lestienne P. Eloy L.. (2014). Design, synthesis and biological evaluation of Pontin ATPase inhibitors through a molecular docking approach. Bioorg. Med. Chem. Lett. 24, 2512–2516. 10.1016/j.bmcl.2014.04.003 24767849

[B46] Etard C. Gradl D. Kunz M. Eilers M. Wedlich D. (2005). Pontin and Reptin regulate cell proliferation in early Xenopus embryos in collaboration with c-Myc and Miz-1. Mech. Dev. 122, 545–556. 10.1016/j.mod.2004.11.010 15804567

[B47] Fielding A. B. Dobreva I. Mcdonald P. C. Foster L. J. Dedhar S. (2008). Integrin-linked kinase localizes to the centrosome and regulates mitotic spindle organization. J. Cell Biol. 180, 681–689. 10.1083/jcb.200710074 18283114 PMC2265580

[B48] Flavin P. Redmond A. Mcbryan J. Cocchiglia S. Tibbitts P. Fahy-Browne P.. (2011). RuvBl2 cooperates with Ets2 to transcriptionally regulate hTERT in colon cancer. FEBS Lett. 585, 2537–2544. 10.1016/j.febslet.2011.07.005 21763315

[B49] Forget D. Lacombe A. A. Cloutier P. Lavallee-Adam M. Blanchette M. Coulombe B. (2013). Nuclear import of RNA polymerase II is coupled with nucleocytoplasmic shuttling of the RNA polymerase II-associated protein 2. Nucleic Acids Res. 41, 6881–6891. 10.1093/nar/gkt455 23723243 PMC3737550

[B50] Gallant P. (2007). Control of transcription by Pontin and Reptin. Trends Cell Biol. 17, 187–192. 10.1016/j.tcb.2007.02.005 17320397

[B51] Gartner W. Rossbacher J. Zierhut B. Daneva T. Base W. Weissel M.. (2003). The ATP-dependent helicase RUVBL1/TIP49a associates with tubulin during mitosis. Cell Motil. Cytoskeleton 56, 79–93. 10.1002/cm.10136 14506706

[B52] Gentili C. Castor D. Kaden S. Lauterbach D. Gysi M. Steigemann P.. (2015). Chromosome missegregation associated with RUVBL1 deficiency. PLoS ONE 10:e0133576. 10.1371/journal.pone.0133576 26201077 PMC4511761

[B53] Gilmore T. D. (2006). Introduction to NF-kappaB: players, pathways, perspectives. Oncogene 25, 6680–6684. 10.1038/sj.onc.1209954 17072321

[B54] Gnatovskiy L. Mita P. Levy D. E. (2013). The human RVB complex is required for efficient transcription of type I interferon-stimulated genes. Mol. Cell. Biol. 33, 3817–3825. 10.1128/MCB.01562-12 23878400 PMC3811876

[B55] Gorynia S. Bandeiras T. M. Pinho F. G. Mcvey C. E. Vonrhein C. Round A.. (2011). Structural and functional insights into a dodecameric molecular machine - the RuvBL1/RuvBL2 complex. J. Struct. Biol. 176, 279–291. 10.1016/j.jsb.2011.09.001 21933716

[B56] Gospodinov A. Vaissiere T. Krastev D. B. Legube G. Anachkova B. Herceg Z. (2011). Mammalian Ino80 mediates double-strand break repair through its role in DNA end strand resection. Mol. Cell. Biol. 31, 4735–4745. 10.1128/MCB.06182-11 21947284 PMC3232918

[B57] Gray T. A. Murray E. Nowicki M. W. Remnant L. Scherl A. Muller P.. (2013). Development of a fluorescent monoclonal antibody-based assay to measure the allosteric effects of synthetic peptides on self-oligomerization of AGR2 protein. Protein Sci. 22, 1266–1278. 10.1002/pro.2299 23780840 PMC3776338

[B58] Grieb B. C. Gramling M. W. Arrate M. P. Chen X. Beauparlant S. L. Haines D. S.. (2014). Oncogenic protein MTBP interacts with MYC to promote tumorigenesis. Cancer Res. 74, 3591–3602. 10.1158/0008-5472.CAN-13-2149 24786788 PMC4079748

[B59] Grigoletto A. Lestienne P. Rosenbaum J. (2011). The multifaceted proteins Reptin and Pontin as major players in cancer. Biochim. Biophys. Acta 1815, 147–157. 10.1016/j.bbcan.2010.11.002 21111787

[B60] Guo C. C. Dadhania V. Zhang L. Majewski T. Bondaruk J. Sykulski M.. (2016). Gene expression profile of the clinically aggressive micropapillary variant of bladder cancer. Eur. Urol. 70, 611–620. 10.1016/j.eururo.2016.02.056 26988609 PMC5804336

[B61] Haurie V. Menard L. Nicou A. Touriol C. Metzler P. Fernandez J.. (2009). Adenosine triphosphatase pontin is overexpressed in hepatocellular carcinoma and coregulated with reptin through a new posttranslational mechanism. Hepatology 50, 1871–1883. 10.1002/hep.23215 19877184 PMC2927003

[B62] Herter E. K. Stauch M. Gallant M. Wolf E. Raabe T. Gallant P. (2015). snoRNAs are a novel class of biologically relevant Myc targets. BMC Biol. 13:25. 10.1186/s12915-015-0132-6 25888729 PMC4430873

[B63] Horejsi Z. Stach L. Flower T. G. Joshi D. Flynn H. Skehel J. M.. (2014). Phosphorylation-dependent PIH1D1 interactions define substrate specificity of the R2TP cochaperone complex. Cell Rep. 7, 19–26. 10.1016/j.celrep.2014.03.013 24656813 PMC3989777

[B64] Horejsi Z. Takai H. Adelman C. A. Collis S. J. Flynn H. Maslen S.. (2010). CK2 phospho-dependent binding of R2TP complex to TEL2 is essential for mTOR and SMG1 stability. Mol. Cell 39, 839–850. 10.1016/j.molcel.2010.08.037 20864032

[B65] Huber O. Menard L. Haurie V. Nicou A. Taras D. Rosenbaum J. (2008). Pontin and reptin, two related ATPases with multiple roles in cancer. Cancer Res. 68, 6873–6876. 10.1158/0008-5472.CAN-08-0547 18757398

[B66] Hustedt N. Durocher D. (2016). The control of DNA repair by the cell cycle. Nat. Cell Biol. 19, 1–9. 10.1038/ncb3452 28008184

[B67] Ikura T. Tashiro S. Kakino A. Shima H. Jacob N. Amunugama R.. (2007). DNA damage-dependent acetylation and ubiquitination of H2AX enhances chromatin dynamics. Mol. Cell. Biol. 27, 7028–7040. 10.1128/MCB.00579-07 17709392 PMC2168918

[B68] Izumi N. Yamashita A. Iwamatsu A. Kurata R. Nakamura H. Saari B.. (2010). AAA+ proteins RUVBL1 and RUVBL2 coordinate PIKK activity and function in nonsense-mediated mRNA decay. Sci. Signal. 3:ra27. 10.1126/scisignal.2000468 20371770

[B69] Jacquet K. Fradet-Turcotte A. Avvakumov N. Lambert J. P. Roques C. Pandita R. K.. (2016). The TIP60 complex regulates bivalent chromatin recognition by 53BP1 through direct H4K20me binding and H2AK15 acetylation. Mol. Cell 62, 409–421. 10.1016/j.molcel.2016.03.031 27153538 PMC4887106

[B70] Jeggo P. A. Pearl L. H. Carr A. M. (2016). DNA repair, genome stability and cancer: a historical perspective. Nat. Rev. Cancer 16, 35–42. 10.1038/nrc.2015.4 26667849

[B71] Jeong Y. Y. Her J. Oh S. Y. Chung I. K. (2016). Hsp90-binding immunophilin FKBP52 modulates telomerase activity by promoting the cytoplasmic retrotransport of hTERT. Biochem. J. 473, 3517–3532. 10.1042/BCJ20160344 27503910

[B72] Jeronimo C. Forget D. Bouchard A. Li Q. Chua G. Poitras C.. (2007). Systematic analysis of the protein interaction network for the human transcription machinery reveals the identity of the 7SK capping enzyme. Mol. Cell 27, 262–274. 10.1016/j.molcel.2007.06.027 17643375 PMC4498903

[B73] Jha S. Dutta A. (2009). RVB1/RVB2: running rings around molecular biology. Mol. Cell 34, 521–533. 10.1016/j.molcel.2009.05.016 19524533 PMC2733251

[B74] Jha S. Gupta A. Dar A. Dutta A. (2013). RVBs are required for assembling a functional TIP60 complex. Mol. Cell. Biol. 33, 1164–1174. 10.1128/MCB.01567-12 23297341 PMC3592018

[B75] Jha S. Shibata E. Dutta A. (2008). Human Rvb1/Tip49 is required for the histone acetyltransferase activity of Tip60/NuA4 and for the downregulation of phosphorylation on H2AX after DNA damage. Mol. Cell. Biol. 28, 2690–2700. 10.1128/MCB.01983-07 18285460 PMC2293106

[B76] Johnson J. Thijssen B. Mcdermott U. Garnett M. Wessels L. F. Bernards R. (2016). Targeting the RB-E2F pathway in breast Cancer 35, 4829–4835. 10.1038/onc.2016.32 26923330 PMC4950965

[B77] Johnston J. A. Ward C. L. Kopito R. R. (1998). Aggresomes: a cellular response to misfolded proteins. J. Cell Biol. 143, 1883–1898. 10.1083/jcb.143.7.1883 9864362 PMC2175217

[B78] Kakihara Y. Saeki M. (2014). The R2TP chaperone complex: its involvement in snoRNP assembly and tumorigenesis. Biomol. Concepts 5, 513–520. 10.1515/bmc-2014-0028 25429602

[B79] Kakihara Y. Makhnevych T. Zhao L. Tang W. Houry W. A. (2014). Nutritional status modulates box C/D snoRNP biogenesis by regulated subcellular relocalization of the R2TP complex. Genome Biol. 15:404. 10.1186/s13059-014-0404-4 25060708 PMC4165372

[B80] Kamano Y. Saeki M. Egusa H. Kakihara Y. Houry W. A. Yatani H.. (2013). PIH1D1 interacts with mTOR complex 1 and enhances ribosome RNA transcription. FEBS Lett. 587, 3303–3308. 10.1016/j.febslet.2013.09.001 24036451

[B81] Kim J. H. Choi H. J. Kim B. Kim M. H. Lee J. M. Kim I. S.. (2006). Roles of sumoylation of a reptin chromatin-remodelling complex in cancer metastasis. Nat. Cell Biol. 8, 631–639. 10.1038/ncb1415 16699503

[B82] Kim J. H. Gurumurthy C. B. Naramura M. Zhang Y. Dudley A. T. Doglio L.. (2009). Role of mammalian Ecdysoneless in cell cycle regulation. J. Biol. Chem. 284, 26402–26410. 10.1074/jbc.M109.030551 19640839 PMC2785328

[B83] Kim J. H. Kim B. Cai L. Choi H. J. Ohgi K. A. Tran C.. (2005). Transcriptional regulation of a metastasis suppressor gene by Tip60 and beta-catenin complexes. Nature 434, 921–926. 10.1038/nature03452 15829968

[B84] Kim J. H. Lee J. M. Nam H. J. Choi H. J. Yang J. W. Lee J. S.. (2007). SUMOylation of pontin chromatin-remodeling complex reveals a signal integration code in prostate cancer cells. Proc. Natl. Acad. Sci. U.S.A. 104, 20793–20798. 10.1073/pnas.0710343105 18087039 PMC2410081

[B85] Kim S. G. Hoffman G. R. Poulogiannis G. Buel G. R. Jang Y. J. Lee K. W.. (2013). Metabolic stress controls mTORC1 lysosomal localization and dimerization by regulating the TTT-RUVBL1/2 complex. Mol. Cell 49, 172–185. 10.1016/j.molcel.2012.10.003 23142078 PMC3545014

[B86] Kurokawa Y. Kanemaki M. Makino Y. Tamura T. A. (1999). A notable example of an evolutionary conserved gene: studies on a putative DNA helicase TIP49. DNA Seq. 10, 37–42. 10.3109/10425179909033934 10565543

[B87] Kusch T. Florens L. Macdonald W. H. Swanson S. K. Glaser R. L. Yates J. R. III.. (2004). Acetylation by Tip60 is required for selective histone variant exchange at DNA lesions. Science 306, 2084–2087. 10.1126/science.1103455 15528408

[B88] Lacombe J. Mange A. Bougnoux A. C. Prassas I. Solassol J. (2014). A multiparametric serum marker panel as a complementary test to mammography for the diagnosis of node-negative early-stage breast cancer and DCIS in young women. Cancer Epidemiol. Biomarkers Prev. 23, 1834–1842. 10.1158/1055-9965.EPI-14-0267 24957886

[B89] Lacombe J. Mange A. Jarlier M. Bascoul-Mollevi C. Rouanet P. Lamy P. J.. (2013). Identification and validation of new autoantibodies for the diagnosis of DCIS and node negative early-stage breast cancers. Int. J. Cancer 132, 1105–1113. 10.1002/ijc.27766 22886747

[B90] Lamouille S. Xu J. Derynck R. (2014). Molecular mechanisms of epithelial-mesenchymal transition. Nat. Rev. Mol. Cell Biol. 15, 178–196. 10.1038/nrm3758 24556840 PMC4240281

[B91] Lauscher J. C. Elezkurtaj S. Dullat S. Lipka S. Grone J. Buhr H. J.. (2012). Increased Pontin expression is a potential predictor for outcome in sporadic colorectal carcinoma. Oncol. Rep. 28, 1619–1624. 10.3892/or.2012.1968 22895545

[B92] Lauscher J. C. Loddenkemper C. Kosel L. Grone J. Buhr H. J. Huber O. (2007). Increased pontin expression in human colorectal cancer tissue. Hum. Pathol. 38, 978–985. 10.1016/j.humpath.2007.01.005 17442372

[B93] Lee J. H. Chung I. K. (2010). Curcumin inhibits nuclear localization of telomerase by dissociating the Hsp90 co-chaperone p23 from hTERT. Cancer Lett. 290, 76–86. 10.1016/j.canlet.2009.08.026 19751963

[B94] Lee J. S. Kim Y. Bhin J. Shin H. J. Nam H. J. Lee S. H.. (2011). Hypoxia-induced methylation of a pontin chromatin remodeling factor. Proc. Natl. Acad. Sci. U.S.A. 108, 13510–13515. 10.1073/pnas.1106106108 21825155 PMC3158161

[B95] Lee J. S. Kim Y. Kim I. S. Kim B. Choi H. J. Lee J. M.. (2010). Negative regulation of hypoxic responses via induced Reptin methylation. Mol. Cell 39, 71–85. 10.1016/j.molcel.2010.06.008 20603076 PMC4651011

[B96] Li W. Zeng J. Li Q. Zhao L. Liu T. Bjorkholm M.. (2010). Reptin is required for the transcription of telomerase reverse transcriptase and over-expressed in gastric cancer. Mol. Cancer 9:132. 10.1186/1476-4598-9-132 20509972 PMC2887797

[B97] Li Y. Tergaonkar V. (2014). Noncanonical functions of telomerase: implications in telomerase-targeted cancer therapies. Cancer Res. 74, 1639–1644. 10.1158/0008-5472.CAN-13-3568 24599132

[B98] Liao D. J. Thakur A. Wu J. Biliran H. Sarkar F. H. (2007). Perspectives on c-Myc, Cyclin D1, and their interaction in cancer formation, progression, and response to chemotherapy. Crit. Rev. Oncog. 13, 93–158. 10.1615/CritRevOncog.v13.i2.10 18197790

[B99] Lopez-Perrote A. Alatwi H. E. Torreira E. Ismail A. Ayora S. Downs J. A.. (2014). Structure of Yin Yang 1 oligomers that cooperate with RuvBL1-RuvBL2 ATPases. J. Biol. Chem. 289, 22614–22629. 10.1074/jbc.M114.567040 24990942 PMC4132769

[B100] Lottaz D. Maurer C. A. Noel A. Blacher S. Huguenin M. Nievergelt A.. (2011). Enhanced activity of meprin-alpha, a pro-migratory and pro-angiogenic protease, in colorectal cancer. PLoS ONE 6:e26450. 10.1371/journal.pone.0026450 22096485 PMC3214016

[B101] Lui L. Lowe T. (2013). Small nucleolar RNAs and RNA-guided post-transcriptional modification. Essays Biochem. 54, 53–77. 10.1042/bse0540053 23829527

[B102] Macdonald B. T. Tamai K. He X. (2009). Wnt/beta-catenin signaling: components, mechanisms, and diseases. Dev. Cell 17, 9–26. 10.1016/j.devcel.2009.06.016 19619488 PMC2861485

[B103] Machado-Pinilla R. Liger D. Leulliot N. Meier U. T. (2012). Mechanism of the AAA+ ATPases pontin and reptin in the biogenesis of H/ACA RNPs. RNA 18, 1833–1845. 10.1261/rna.034942.112 22923768 PMC3446707

[B104] Maciejowski J. de Lange T. (2017). Telomeres in cancer: tumour suppression and genome instability. Nat. Rev. Mol. Cell Biol. 18, 175–186. 10.1038/nrm.2016.171 28096526 PMC5589191

[B105] Magalska A. Schellhaus A. K. Moreno-Andres D. Zanini F. Schooley A. Sachdev R.. (2014). RuvB-like ATPases function in chromatin decondensation at the end of mitosis. Dev. Cell 31, 305–318. 10.1016/j.devcel.2014.09.001 25443297

[B106] Makino Y. Mimori T. Koike C. Kanemaki M. Kurokawa Y. Inoue S.. (1998). TIP49, homologous to the bacterial DNA helicase RuvB, acts as an autoantigen in human. Biochem. Biophys. Res. Commun. 245, 819–823. 10.1006/bbrc.1998.8504 9588198

[B107] Malinova A. Cvackova Z. Mateju D. Horejsi Z. Abeza C. Vandermoere F.. (2017). Assembly of the U5 snRNP component PRPF8 is controlled by the HSP90/R2TP chaperones. J. Cell Biol. 216, 1579–1596. 10.1083/jcb.201701165 28515276 PMC5461031

[B108] Malumbres M. Barbacid M. (2001). To cycle or not to cycle: a critical decision in cancer. Nat. Rev. Cancer 1, 222–231. 10.1038/35106065 11902577

[B109] Mannoor K. Liao J. Jiang F. (2012). Small nucleolar RNAs in cancer. Biochim. Biophys. Acta 1826, 121–128. 10.1016/j.bbcan.2012.03.005 22498252 PMC3842010

[B110] Marcucci F. Stassi G. De Maria R. (2016). Epithelial-mesenchymal transition: a new target in anticancer drug discovery. Nat. Rev. Drug Discov. 15, 311–325. 10.1038/nrd.2015.13 26822829

[B111] Markossian K. A. Kurganov B. I. (2004). Protein folding, misfolding, and aggregation. Formation of inclusion bodies and aggresomes. Biochemistry 69, 971–984. 10.1023/B:BIRY.0000043539.07961.4c 15521811

[B112] Maslon M. M. Hrstka R. Vojtesek B. Hupp T. R. (2010). A divergent substrate-binding loop within the pro-oncogenic protein anterior gradient-2 forms a docking site for Reptin. J. Mol. Biol. 404, 418–438. 10.1016/j.jmb.2010.09.035 20888340

[B113] Massenet S. Bertrand E. Verheggen C. (2016). Assembly and trafficking of box C/D and H/ACA snoRNPs. RNA Biol. 4, 680–692. 10.1080/15476286.2016.1243646 PMC5519232 27715451

[B114] Matera A. G. Wang Z. (2014). A day in the life of the spliceosome. Nat. Rev. Mol. Cell Biol. 15, 108–121. 10.1038/nrm3742 24452469 PMC4060434

[B115] Matias P. M. Baek S. H. Bandeiras T. M. Dutta A. Houry W. A. Llorca O.. (2015). The AAA+ proteins Pontin and Reptin enter adult age: from understanding their basic biology to the identification of selective inhibitors. Front. Mol. Biosci. 2:17. 10.3389/fmolb.2015.00017 25988184 PMC4428354

[B116] Matias P. M. Gorynia S. Donner P. Carrondo M. A. (2006). Crystal structure of the human AAA+ protein RuvBL1. J. Biol. Chem. 281, 38918–38929. 10.1074/jbc.M605625200 17060327

[B117] McCubrey J. A. Rakus D. Gizak A. Steelman L. S. Abrams S. L. Lertpiriyapong K.. (2016). Effects of mutations in Wnt/beta-catenin, hedgehog, Notch and PI3K pathways on GSK-3 activity-Diverse effects on cell growth, metabolism and cancer. Biochim. Biophys. Acta 1863, 2942–2976. 10.1016/j.bbamcr.2016.09.004 27612668

[B118] McKeegan K. S. Debieux C. M. Watkins N. J. (2009). Evidence that the AAA+ proteins TIP48 and TIP49 bridge interactions between 15.5K and the related NOP56 and NOP58 proteins during box C/D snoRNP biogenesis. Mol. Cell. Biol. 29, 4971–4981. 10.1128/MCB.00752-09 19620283 PMC2738292

[B119] McKeegan K. S. Debieux C. M. Boulon S. Bertrand E. Watkins N. J. (2007). A dynamic scaffold of pre-snoRNP factors facilitates human box C/D snoRNP assembly. Mol. Cell. Biol. 27, 6782–6793. 10.1128/MCB.01097-07 17636026 PMC2099223

[B120] Mei Y. P. Liao J. P. Shen J. Yu L. Liu B. L. Liu L.. (2012). Small nucleolar RNA 42 acts as an oncogene in lung tumorigenesis. Oncogene 31, 2794–2804. 10.1038/onc.2011.449 21986946 PMC4966663

[B121] Menard L. Taras D. Grigoletto A. Haurie V. Nicou A. Dugot-Senant N.. (2010). *In vivo* silencing of Reptin blocks the progression of human hepatocellular carcinoma in xenografts and is associated with replicative senescence. J. Hepatol. 52, 681–689. 10.1016/j.jhep.2009.12.029 20346530

[B122] Millan-Zambrano G. Chavez S. (2014). Nuclear functions of prefoldin. Open Biol 4:140085. 10.1098/rsob.140085 25008233 PMC4118604

[B123] Milone M. R. Pucci B. Colangelo T. Lombardi R. Iannelli F. Colantuoni V.. (2016). Proteomic characterization of peroxisome proliferator-activated receptor-gamma (PPARgamma) overexpressing or silenced colorectal cancer cells unveils a novel protein network associated with an aggressive phenotype. Mol. Oncol. 10, 1344–1362. 10.1016/j.molonc.2016.07.006 27499265 PMC5423198

[B124] Minder P. Bayha E. Becker-Pauly C. Sterchi E. E. (2012). Meprinalpha transactivates the epidermal growth factor receptor (EGFR) via ligand shedding, thereby enhancing colorectal cancer cell proliferation and migration. J. Biol. Chem. 287, 35201–35211. 10.1074/jbc.M112.368910 22923609 PMC3471737

[B125] Mir R. A. Bele A. Mirza S. Srivastava S. Olou A. A. Ammons S. A.. (2015). A novel interaction of Ecdysoneless (ECD) protein with R2TP complex component RUVBL1 is required for the functional role of ECD in cell cycle progression. Mol. Cell. Biol. 36, 886–899. 10.1128/MCB.00594-15 26711270 PMC4810467

[B126] Mita P. Savas J. N. Ha S. Djouder N. Yates J. R. III. Logan S. K. (2013). Analysis of URI nuclear interaction with RPB5 and components of the R2TP/prefoldin-like complex. PLoS ONE 8:e63879. 10.1371/journal.pone.0063879 23667685 PMC3648552

[B127] Mizuno K. Tokumasu A. Nakamura A. Hayashi Y. Kojima Y. Kohri K.. (2006). Genes associated with the formation of germ cells from embryonic stem cells in cultures containing different glucose concentrations. Mol. Reprod. Dev. 73, 437–445. 10.1002/mrd.20395 16425234

[B128] Montanaro L. Trere D. Derenzini M. (2008). Nucleolus, ribosomes, and cancer. Am. J. Pathol. 173, 301–310. 18583314 10.2353/ajpath.2008.070752 PMC2475768

[B129] Morin P. J. (1999). beta-catenin signaling and cancer. Bioessays 21, 1021–1030. 10.1002/(SICI)1521-1878(199912)22:1<1021::AID-BIES6>3.0.CO;2-P 10580987

[B130] Moynagh P. N. (2005). The NF-kappaB pathway. J. Cell Sci. 118, 4589–4592. 10.1242/jcs.02579 16219681

[B131] Muller P. A. Vousden K. H. (2013). p53 mutations in cancer. Nat. Cell Biol. 15, 2–8. 10.1038/ncb2641 23263379

[B132] Nano N. Houry W. A. (2013). Chaperone-like activity of the AAA+ proteins Rvb1 and Rvb2 in the assembly of various complexes. Philos. Trans. R. Soc. Lond. B. Biol. Sci. 368:20110399. 10.1098/rstb.2011.0399 23530256 PMC3638392

[B133] Nguyen T. H. D. Galej W. P. Bai X.-C. Savva C. G. Newman A. J. Scheres S. H. W.. (2015). The architecture of the spliceosomal U4/U6.U5 tri-snRNP. Nature 523, 47–52. 10.1038/nature14548 26106855 PMC4536768

[B134] Ni L. Saeki M. Xu L. Nakahara H. Saijo M. Tanaka K.. (2009). RPAP3 interacts with Reptin to regulate UV-induced phosphorylation of H2AX and DNA damage. J. Cell. Biochem. 106, 920–928. 10.1002/jcb.22073 19180575

[B135] Niewiarowski A. Bradley A. S. Gor J. Mckay A. R. Perkins S. J. Tsaneva I. R. (2010). Oligomeric assembly and interactions within the human RuvB-like RuvBL1 and RuvBL2 complexes. Biochem. J. 429, 113–125. 10.1042/BJ20100489 20412048

[B136] Ocak S. Friedman D. B. Chen H. Ausborn J. A. Hassanein M. Detry B.. (2014). Discovery of new membrane-associated proteins overexpressed in small-cell lung cancer. J. Thorac. Oncol. 9, 324–336. 10.1097/JTO.0000000000000090 24518087 PMC4028683

[B137] O'Connor M. J. (2015). Targeting the DNA damage response in cancer. Mol. Cell 60, 547–560. 10.1016/j.molcel.2015.10.040 26590714

[B138] Osaki H. Walf-Vorderwulbecke V. Mangolini M. Zhao L. Horton S. J. Morrone G.. (2013). The AAA+ ATPase RUVBL2 is a critical mediator of MLL-AF9 oncogenesis. Leukemia 27, 1461–1468. 10.1038/leu.2013.42 23403462

[B139] Otsuji N. Iyehara H. Hideshima Y. (1974). Isolation and characterization of an *Escherichia coli* ruv mutant which forms nonseptate filaments after low doses of ultraviolet light irradiation. J. Bacteriol. 117, 337–344. 4590461 10.1128/jb.117.2.337-344.1974 PMC285519

[B140] Otto T. Sicinski P. (2017). Cell cycle proteins as promising targets in cancer therapy. Nat. Rev. Cancer 17, 93–115. 10.1038/nrc.2016.138 28127048 PMC5345933

[B141] Ozenne P. Eymin B. Brambilla E. Gazzeri S. (2010). The ARF tumor suppressor: structure, functions and status in cancer. Int. J. Cancer 127, 2239–2247. 10.1002/ijc.25511 20549699

[B142] Pal M. Morgan M. Phelps S. E. Roe S. M. Parry-Morris S. Downs J. A.. (2014). Structural basis for phosphorylation-dependent recruitment of Tel2 to Hsp90 by Pih1. Structure 22, 805–818. 10.1016/j.str.2014.04.001 24794838 PMC4058522

[B143] Perez-Perri J. I. Dengler V. L. Audetat K. A. Pandey A. Bonner E. A. Urh M.. (2016). The TIP60 complex is a conserved coactivator of HIF1A. Cell Rep. 16, 37–47. 10.1016/j.celrep.2016.05.082 27320910 PMC4957981

[B144] Petronczki M. Lenart P. Peters J. M. (2008). Polo on the rise-from mitotic entry to cytokinesis with Plk1. Dev. Cell 14, 646–659. 10.1016/j.devcel.2008.04.014 18477449

[B145] Prieto M. B. Georg R. C. Gonzales-Zubiate F. A. Luz J. S. Oliveira C. C. (2015). Nop17 is a key R2TP factor for the assembly and maturation of box C/D snoRNP complex. BMC Mol. Biol. 16:7. 10.1186/s12867-015-0037-5 25888478 PMC4377001

[B146] Prosser S. L. Pelletier L. (2017). Mitotic spindle assembly in animal cells: a fine balancing act. Nat. Rev. Mol. Cell Biol. 18, 187–201. 10.1038/nrm.2016.162 28174430

[B147] Qiu H. Gao Y. Mao D. (2015). Reptin physically interacts with p65 and represses NF-kappaB activation. FEBS Lett. 589, 1951–1957. 10.1016/j.febslet.2015.04.028 25957047

[B148] Queval R. Papin C. Dalvai M. Bystricky K. Humbert O. (2014). Reptin and Pontin oligomerization and activity are modulated through histone H3 N-terminal tail interaction. J. Biol. Chem. 289, 33999–34012. 10.1074/jbc.M114.576785 25336637 PMC4256336

[B149] Rajendra E. Garaycoechea J. I. Patel K. J. Passmore L. A. (2014). Abundance of the Fanconi anaemia core complex is regulated by the RuvBL1 and RuvBL2 AAA+ ATPases. Nucleic Acids Res. 42, 13736–13748. 10.1093/nar/gku1230 25428364 PMC4267650

[B150] Rashid S. Pilecka I. Torun A. Olchowik M. Bielinska B. Miaczynska M. (2009). Endosomal adaptor proteins APPL1 and APPL2 are novel activators of beta-catenin/TCF-mediated transcription. J. Biol. Chem. 284, 18115–18128. 10.1074/jbc.M109.007237 19433865 PMC2709337

[B151] Raymond A. A. Benhamouche S. Neaud V. Di Martino J. Javary J. Rosenbaum J. (2015). Reptin regulates DNA double strand breaks repair in human hepatocellular carcinoma. PLoS ONE 10:e0123333. 10.1371/journal.pone.0123333 25875766 PMC4398330

[B152] Ren J. Li W. Liu H. Yan L. Jiao W. Li D.. (2013). Overexpression of reptin in renal cell carcinoma contributes to tumor malignancies and its inhibition triggers senescence of cancer cells. Urol. Oncol. 31, 1358–1366. 10.1016/j.urolonc.2012.01.004 22341977

[B153] Rosenbaum J. Baek S. H. Dutta A. Houry W. A. Huber O. Hupp T. R.. (2013). The emergence of the conserved AAA+ ATPases Pontin and Reptin on the signaling landscape. Sci. Signal. 6:mr1. 10.1126/scisignal.2003906 23482663 PMC4201591

[B154] Rousseau B. Menard L. Haurie V. Taras D. Blanc J. F. Moreau-Gaudry F.. (2007). Overexpression and role of the ATPase and putative DNA helicase RuvB-like 2 in human hepatocellular carcinoma. Hepatology 46, 1108–1118. 10.1002/hep.21770 17657734

[B155] Rowe A. Weiske J. Kramer T. S. Huber O. Jackson P. (2008). Phorbol ester enhances KAI1 transcription by recruiting Tip60/pontin complexes. Neoplasia 10, 1421–1432. 10.1593/neo.08850 19048121 PMC2586693

[B156] Ruggero D. Pandolfi P. P. (2003). Does the ribosome translate cancer? Nat. Rev. Cancer 3, 179–192. 10.1038/nrc1015 12612653

[B157] Scheidereit C. (2006). IkappaB kinase complexes: gateways to NF-kappaB activation and transcription. Oncogene 25, 6685–6705. 10.1038/sj.onc.1209934 17072322

[B158] Sherr C. J. Weber J. D. (2000). The ARF/p53 pathway. Curr. Opin. Genet. Dev. 10, 94–99. 10.1016/S0959-437X(99)00038-6 10679383

[B159] Shi G. Jin Y. (2010). Role of Oct4 in maintaining and regaining stem cell pluripotency. Stem Cell Res. Ther. 1:39. 10.1186/scrt39 21156086 PMC3025441

[B160] Shimada K. Saeki M. Egusa H. Fukuyasu S. Yura Y. Iwai K.. (2011). RPAP3 enhances cytotoxicity of doxorubicin by impairing NF-kappa B pathway. Biochem. Biophys. Res. Commun. 404, 910–914. 10.1016/j.bbrc.2010.12.071 21184742

[B161] Shtutman M. Zhurinsky J. Simcha I. Albanese C. D'amico M. Pestell R.. (1999). The cyclin D1 gene is a target of the beta-catenin/LEF-1 pathway. Proc. Natl. Acad. Sci. U.S.A. 96, 5522–5527. 10.1073/pnas.96.10.5522 10318916 PMC21892

[B162] Si J. Yu X. Zhang Y. Dewille J. W. (2010). Myc interacts with Max and Miz1 to repress C/EBPdelta promoter activity and gene expression. Mol. Cancer 9:92. 10.1186/1476-4598-9-92 20426839 PMC2879254

[B163] Sigala B. Edwards M. Puri T. Tsaneva I. R. (2005). Relocalization of human chromatin remodeling cofactor TIP48 in mitosis. Exp. Cell Res. 310, 357–369. 10.1016/j.yexcr.2005.07.030 16157330

[B164] Su H. Xu T. Ganapathy S. Shadfan M. Long M. Huang T. H.. (2014). Elevated snoRNA biogenesis is essential in breast cancer. Oncogene 33, 1348–1358. 10.1038/onc.2013.89 23542174

[B165] Sun Y. Jiang X. Chen S. Fernandes N. Price B. D. (2005). A role for the Tip60 histone acetyltransferase in the acetylation and activation of ATM. Proc. Natl. Acad. Sci. U.S.A. 102, 13182–13187. 10.1073/pnas.0504211102 16141325 PMC1197271

[B166] Sun Y. Jiang X. Xu Y. Ayrapetov M. K. Moreau L. A. Whetstine J. R.. (2009). Histone H3 methylation links DNA damage detection to activation of the tumour suppressor Tip60. Nat. Cell Biol. 11, 1376–1382. 10.1038/ncb1982 19783983 PMC2783526

[B167] Takai H. Wang R. C. Takai K. K. Yang H. De Lange T. (2007). Tel2 regulates the stability of PI3K-related protein kinases. Cell 131, 1248–1259. 10.1016/j.cell.2007.10.052 18160036

[B168] Tanaka M. Kim Y. M. Lee G. Junn E. Iwatsubo T. Mouradian M. M. (2004). Aggresomes formed by alpha-synuclein and synphilin-1 are cytoprotective. J. Biol. Chem. 279, 4625–4631. 10.1074/jbc.M310994200 14627698

[B169] Tang J. Cho N. W. Cui G. Manion E. M. Shanbhag N. M. Botuyan M. V.. (2013). Acetylation limits 53BP1 association with damaged chromatin to promote homologous recombination. Nat. Struct. Mol. Biol. 20, 317–325. 10.1038/nsmb.2499 23377543 PMC3594358

[B170] Taniuchi K. Furihata M. Iwasaki S. Tanaka K. Shimizu T. Saito M.. (2014). RUVBL1 directly binds actin filaments and induces formation of cell protrusions to promote pancreatic cancer cell invasion. Int. J. Oncol. 44, 1945–1954. 10.3892/ijo.2014.2380 24728183

[B171] Tao D. King J. G. Tweedell R. E. Jost P. J. Boddey J. A. Dinglasan R. R. (2014). The acute transcriptomic and proteomic response of HC-04 hepatoma cells to hepatocyte growth factor and its implications for Plasmodium falciparum sporozoite invasion. Mol. Cell. Proteomics 13, 1153–1164. 10.1074/mcp.M113.035584 24532842 PMC4014276

[B172] Tarangelo A. Lo N. Teng R. Kim E. Le L. Watson D.. (2015). Recruitment of Pontin/Reptin by E2f1 amplifies E2f transcriptional response during cancer progression. Nat. Commun. 6:10028. 10.1038/ncomms10028 26639898 PMC4686657

[B173] Taubert S. Gorrini C. Frank S. R. Parisi T. Fuchs M. Chan H. M.. (2004). E2F-dependent histone acetylation and recruitment of the Tip60 acetyltransferase complex to chromatin in late G1. Mol. Cell. Biol. 24, 4546–4556. 10.1128/MCB.24.10.4546-4556.2004 15121871 PMC400446

[B174] Tergaonkar V. (2006). NFkappaB pathway: a good signaling paradigm and therapeutic target. Int. J. Biochem. Cell Biol. 38, 1647–1653. 10.1016/j.biocel.2006.03.023 16766221

[B175] Tsukuda T. Lo Y. C. Krishna S. Sterk R. Osley M. A. Nickoloff J. A. (2009). INO80-dependent chromatin remodeling regulates early and late stages of mitotic homologous recombination. DNA Repair 8, 360–369. 10.1016/j.dnarep.2008.11.014 19095087

[B176] Tung C. W. Wu M. T. Chen Y. K. Wu C. C. Chen W. C. Li H. P.. (2013). Identification of biomarkers for esophageal squamous cell carcinoma using feature selection and decision tree methods. Sci. World J. 2013:782031. 10.1155/2013/782031 24396308 PMC3875100

[B177] Uribarri M. Hormaeche I. Zalacain R. Lopez-Vivanco G. Martinez A. Nagore D.. (2014). A new biomarker panel in bronchoalveolar lavage for an improved lung cancer diagnosis. J. Thorac. Oncol. 9, 1504–1512. 10.1097/JTO.0000000000000282 25105437

[B178] Velmurugan B. K. Yang H. H. Sung P. J. Weng C. F. (2017). Excavatolide B inhibits nonsmall cell lung cancer proliferation by altering peroxisome proliferator activated receptor gamma expression and PTEN/AKT/NF-Kbeta expression. Environ. Toxicol. 32, 290–301. 10.1002/tox.22235 26790859

[B179] Venteicher A. S. Meng Z. Mason P. J. Veenstra T. D. Artandi S. E. (2008). Identification of ATPases pontin and reptin as telomerase components essential for holoenzyme assembly. Cell 132, 945–957. 10.1016/j.cell.2008.01.019 18358808 PMC2291539

[B180] Verheggen C. Pradet-Balade B. Bertrand E. (2015). SnoRNPs, ZNHIT proteins and the R2TP pathway. Oncotarget 6, 41399–41400. 10.18632/oncotarget.6388 26623726 PMC4747161

[B181] Von Morgen P. Horejsi Z. Macurek L. (2015). Substrate recognition and function of the R2TP complex in response to cellular stress. Front. Genet. 6:69. 10.3389/fgene.2015.00069 25767478 PMC4341119

[B182] Walz S. Lorenzin F. Morton J. Wiese K. E. Von Eyss B. Herold S.. (2014). Activation and repression by oncogenic MYC shape tumour-specific gene expression profiles. Nature 511, 483–487. 10.1038/nature13473 25043018 PMC6879323

[B183] Watkins N. J. Bohnsack M. T. (2012). The box C/D and H/ACA snoRNPs: key players in the modification, processing and the dynamic folding of ribosomal RNA. Wiley Interdiscip. Rev. RNA 3, 397–414. 10.1002/wrna.117 22065625

[B184] Watkins N. J. Lemm I. Ingelfinger D. Schneider C. Hossbach M. Urlaub H.. (2004). Assembly and maturation of the U3 snoRNP in the nucleoplasm in a large dynamic multiprotein complex. Mol. Cell 16, 789–798. 10.1016/j.molcel.2004.11.012 15574333

[B185] Weiske J. Huber O. (2005). The histidine triad protein Hint1 interacts with Pontin and Reptin and inhibits TCF-beta-catenin-mediated transcription. J. Cell Sci. 118, 3117–3129. 10.1242/jcs.02437 16014379

[B186] Weiske J. Huber O. (2006). The histidine triad protein Hint1 triggers apoptosis independent of its enzymatic activity. J. Biol. Chem. 281, 27356–27366. 10.1074/jbc.M513452200 16835243

[B187] Williams G. T. Farzaneh F. (2012). Are snoRNAs and snoRNA host genes new players in cancer? Nat. Rev. Cancer 12, 84–88. 10.1038/nrc3195 22257949

[B188] Wilson W. R. Hay M. P. (2011). Targeting hypoxia in cancer therapy. Nat. Rev. Cancer 11, 393–410. 10.1038/nrc3064 21606941

[B189] Wood M. A. Mcmahon S. B. Cole M. D. (2000). An ATPase/helicase complex is an essential cofactor for oncogenic transformation by c-Myc. Mol. Cell 5, 321–330. 10.1016/S1097-2765(00)80427-X 10882073

[B190] Wu S. Shi Y. Mulligan P. Gay F. Landry J. Liu H.. (2007). A YY1-INO80 complex regulates genomic stability through homologous recombination-based repair. Nat. Struct. Mol. Biol. 14, 1165–1172. 10.1038/nsmb1332 18026119 PMC2754171

[B191] Xie C. Wang W. Yang F. Wu M. Mei Y. (2012). RUVBL2 is a novel repressor of ARF transcription. FEBS Lett. 586, 435–441. 10.1016/j.febslet.2012.01.026 22285491

[B192] Xie X. Chen Y. Xue P. Fan Y. Deng Y. Peng G.. (2009). RUVBL2, a novel AS160-binding protein, regulates insulin-stimulated GLUT4 translocation. Cell Res. 19, 1090–1097. 10.1038/cr.2009.68 19532121

[B193] Yakulov T. Raggioli A. Franz H. Kemler R. (2013). Wnt3a-dependent and -independent protein interaction networks of chromatin-bound beta-catenin in mouse embryonic stem cells. Mol. Cell. Proteomics 12, 1980–1994. 10.1074/mcp.M112.026914 23592333 PMC3708180

[B194] Yang S. Quaresma A. J. Nickerson J. A. Green K. M. Shaffer S. A. Imbalzano A. N.. (2015). Subnuclear domain proteins in cancer cells support the functions of RUNX2 in the DNA damage response. J. Cell Sci. 128, 728–740. 10.1242/jcs.160051 25609707 PMC4327387

[B195] Yang W. S. Moon H. G. Kim H. S. Choi E. J. Yu M. H. Noh D. Y.. (2012). Proteomic approach reveals FKBP4 and S100A9 as potential prediction markers of therapeutic response to neoadjuvant chemotherapy in patients with breast cancer. J. Proteome Res. 11, 1078–1088. 10.1021/pr2008187 22074005

[B196] Yuan X. S. Wang Z. T. Hu Y. J. Bao F. C. Yuan P. Zhang C. (2016). Downregulation of RUVBL1 inhibits proliferation of lung adenocarcinoma cells by G1/S phase cell cycle arrest via multiple mechanisms. Tumour Biol. 37, 16015–16027. 10.1007/s13277-016-5452-9 27722820

[B197] Zaarur N. Xu X. Lestienne P. Meriin A. B. Mccomb M. Costello C. E.. (2015). RuvbL1 and RuvbL2 enhance aggresome formation and disaggregate amyloid fibrils. EMBO J. 34, 2363–2382. 10.15252/embj.201591245 26303906 PMC4570522

[B198] Zhang J. Jiang H. Y. Zhang L. K. Xu W. L. Qiao Y. T. Zhu X. G.. (2017). C-FLIPL modulated Wnt/beta-catenin activation via association with TIP49 protein. J. Biol. Chem. 292, 2132–2142. 10.1074/jbc.M116.753251 28028178 PMC5313088

[B199] Zhang X. Ren J. Yan L. Tang Y. Zhang W. Li D.. (2015). Cytoplasmic expression of pontin in renal cell carcinoma correlates with tumor invasion, metastasis and patients' survival. PLoS ONE 10:e0118659. 10.1371/journal.pone.0118659 25751257 PMC4353622

[B200] Zhao L. J. Loewenstein P. M. Green M. (2016). Ad E1A 243R oncoprotein promotes association of proto-oncogene product MYC with the NuA4/Tip60 complex via the E1A N-terminal repression domain. Virology 499, 178–184. 10.1016/j.virol.2016.09.005 27664947 PMC5109832

[B201] Zhao R. Davey M. Hsu Y. C. Kaplanek P. Tong A. Parsons A. B.. (2005). Navigating the chaperone network: an integrative map of physical and genetic interactions mediated by the hsp90 chaperone. Cell 120, 715–727. 10.1016/j.cell.2004.12.024 15766533

[B202] Zhao Y. Zhang C. Yue X. Li X. Liu J. Yu H.. (2015). Pontin, a new mutant p53-binding protein, promotes gain-of-function of mutant p53. Cell Death Differ. 22, 1824–1836. 10.1038/cdd.2015.33 25857266 PMC4648328

